# Laser-generated Pt/Ni nanocatalysts-carbon nanofibers enabling self-calibrated enzyme-free glucose detection at physiological pH

**DOI:** 10.1007/s00216-025-05869-1

**Published:** 2025-04-11

**Authors:** Christoph Bruckschlegel, Vivien Fleischmann, Aladin Ullrich, Luc Girard, Pierre Bauduin, Antje J. Baeumner, Nongnoot Wongkaew

**Affiliations:** 1 Institute of Analytical Chemistry, Chemo- and Biosensors, University of Regensburg, Universitaetsstrasse 31, 93053 Regensburg, Germany; 2 Institute of Physics, University of Augsburg, Universitaetsstrasse 1, 86159 Augsburg, Germany; 3 ICSM, Univ Montpellier, CEA, CNRS, ENSCM, Marcoule, 30207 Bagnols sur Cèze Cedex, France

**Keywords:** Enzyme-free sensors, Pt/Ni nanocatalysts, Laser-induced carbon nanofibers, Glucose, Physiological pH, Locally generated hydroxide

## Abstract

We propose a bimetallic alloy composed of Pt and Ni embedded within laser-induced carbon nanofibers (Pt/Ni-LCNFs) as an enzyme-free transducer for the detection of glucose under physiological pH. Laser exposure on electrospun polyimide nanofibers, embedded with Pt and Ni precursors, facilitated not only the formation of LCNFs but also the generation of Pt/Ni nanoparticles with a radius of approximately 2 nm and a distinctive crystalline structure. X-ray photoelectron spectroscopy revealed the oxidation states of the laser-generated Pt/Ni and confirmed the formation of the Pt/Ni alloy nanocatalysts. Additionally, small-angle X-ray scattering has shown that the graphitic structures of the LCNFs strongly depend on the metal salt concentrations and molar ratio. Pt/Ni-LCNFs were exploited as enzyme-free electrodes for glucose sensing at physiological pH. The presence of Pt in the alloy enabled a low potential (−0.9 V for 20 s) in situ generation of highly localized OH^−^ which facilitated glucose electrooxidation by Ni. Under optimized conditions, Pt/Ni-LCNFs achieved reliable glucose detection in physiological conditions (pH 7.4), with detection limit of 0.3 mM, linearity from 0.1 to 4 mM, and minimal interference from other electroactive species. Self-calibrated data acquisition strategy provided an excellent recovery rate (95 ± 10%) in diluted human serum. Furthermore, unlike enzyme-based sensors, the catalytic activity of Pt/Ni LCNFs was maintained after sterilization, highlighting their robustness and potential in biomedical applications and bioprocess monitoring.

**Graphical Abstract:**

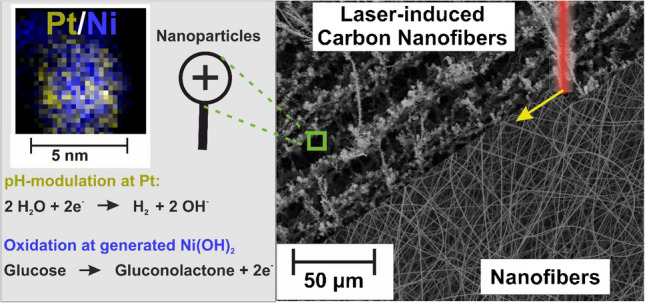

**Supplementary Information:**

The online version contains supplementary material available at 10.1007/s00216-025-05869-1.

## Introduction

Electrochemical sensor–based enzyme-free approaches have shown superior features in terms of cost and stability when compared to conventional enzyme-based sensing strategies. Various kinds of metal and metal oxide nanomaterials have been proposed as useful nanocatalysts in this endeavor, but bimetallic ones have gained significant interest nowadays because they offer synergistic effects that typically make the electrocatalytic reaction better than their monometallic counterparts [1]. In particular, Pt/Ni nanocatalysts have received a lot of attention in the development of enzyme-free sensors. For example, Pt/Ni nanocatalysts are highly attractive for glucose sensing either in alkaline or physiological pH [2–4]. When using Pt alone, the sensors typically suffer from surface poisoning of the nanocatalyst. The addition of a non-noble metal to the nanocatalyst can potentially remove the poisonous intermediates and thus promote electron-transfer reactions [5]. Pt/Ni alloy–decorated multi-walled carbon nanotubes (MWCNTs) have been successfully employed for glucose detection at physiological pH [6]. The Pt/Ni alloys have also been proven to be biocompatible [7, 8], making them highly attractive for biomedical applications.

Various approaches have been reported for synthesis of Pt/Ni alloys or with carbon hybrids and have been successfully applied to enzyme-free sensors. These include chemical reduction [6, 9], electrodeposition [2, 10], and thermal treatment [11]. Despite the great success of the previous reports on synthesis and using Pt/Ni nanocatalysts in enzyme-free sensors, their utility in point-of-care (POC) testing is yet to be achieved because the proposed fabrication techniques are sophisticated, and incompatible for scaling up, and rather costly. Moreover, the measurements mostly require laboratory equipment, e.g., a stirrer, to achieve the greatest analytical performance.

Carbon nanomaterials and their functional hybrids fabricated by laser-writing hold great promise in addressing the aforementioned challenges [12]. Here, the mixture of carbon and metal nanocatalyst precursors can be facilely and economically converted into nanocatalyst-carbon hybrids via CO_2_ laser exposure, as shown in the pioneered work by Tour’s group, in which the as-generated electrodes were mainly used for the oxygen reduction reaction applicable in energy conversion and storage [13]. Nevertheless, their proposed strategy inevitably requires annealing of the precursor substrate prior to the laser exposure. Also, the formation of mixed metal precursors has not yet been investigated [13]. These issues leave room for further studies, in particular towards the manufacturing of enzyme-free sensors in which mass production capability, arbitrary electrode designs, and low overall cost are advantageous.

Our group has employed laser pyrolysis technology to generate 3D-porous carbon electrodes from electrospun polyimide (PI) nanofiber substrates, termed laser-induced carbon nanofibers (LCNFs). Here, unlike the polyamic acid (PAA) which was used by Tour’s group [13], a solvent-soluble PI, Matrimid®5218, has been employed. This thus eliminates the need for an imidization process, i.e., PAA being converted into PI prior to annealing and laser-pyrolysis. Upon optimization of fabrication process parameters [14], the LCNFs exhibit 3D-porous structures and superior analytical performance for common redox markers in comparison to commercially available screen-printed electrodes. We later demonstrated the integration of the LCNF electrodes within miniaturized analytical systems where the devices were able to detect dopamine [15], and DNA targets in pM range [16]. This is clearly attributed to the presence of a 3D-porous nanostructure within the microfluidic analytical system.

Furthermore, we have proven that metal salt precursors, e.g., Ni acetylacetonate, doped within electrospun PI nanofibers can be in situ converted into Ni nanocatalysts embedded within the LCNFs [17]. The as-generated Ni-LCNFs have been successfully applied for glucose detection under alkaline conditions, providing a limit of detection (LOD) in the sub μM range. We also proved that the stability of the Ni nanocatalysts embedded within LCNFs under stirred solution for a long period is much greater than that of electrodeposited Ni onto LCNFs. Recently, we attempted to fabricate bimetallic Pt/Ni alloys embedded within LCNFs where optimization of fabrication processes and electrochemical performance were assessed for H_2_O_2_ as a model analyte [18]. However, the studies mostly focused on analytical aspects where in-depth investigations on the Pt/Ni-LCNFs have not yet explored.

In addition, unlike Ni-LCNFs that require alkaline detection medium for the measurement [17], the Pt/Ni-LCNFs are highly promising for glucose detection under physiological pH. Here, noble metals such as Au, Pd, and Pt have been exploited for in situ generation of OH^−^ via electrochemical reduction of proton (water splitting), subsequently promoting electrocatalytic oxidation of glucose by the catalyst itself [19] or the added active one, e.g., Co or Ni [19, 20]. Despite their excellent analytical performance and potential for integration into wearable glucose sensing devices, the studies still presented some shortcomings. These include the need for complex fabrication systems and high reduction potentials in OH^−^ generation [20, 21]. Moreover, the nanocatalysts must be coated with a polymer layer to ensure mechanical stability after electrodeposition [20] which could diminish their electrocatalytic activity and possibly explain the need for higher reduction potential. Taken together, the beneficial features of Pt/Ni-LCNFs could help address these issues, ultimately leading to highly practical and efficient sensors at lower cost.

We thus aimed to fabricate Pt/Ni-LCNFs and explore their associated electroanalytical performance for enzyme-free glucose detection at physiological pH. We initially characterized the materials by transmission electron microscopy (TEM) and high spatial resolution TEM (HRTEM) to explore the sizes and crystallinity of the nanocatalysts, respectively. Furthermore, we elucidated the Pt/Ni alloyed structure via scanning TEM coupled with energy-dispersive X-ray spectrometry (STEM-EDS). We also revealed the influence of Pt and/or Ni on the resulting graphite/graphene oxide structure of LCNFs and the oxidation states of added metal(s) via X-ray photoelectron spectroscopy (XPS). Additionally, small-angle X-ray scattering (SAXS) was used to characterize the graphitic structure of the LCNFs, specifically the stacking of graphene sheets and their interlamellar distance. The capability of Pt/Ni nanocatalysts to generate local OH^−^ and subsequently oxidize glucose, as well as the influence of their composition, was carefully examined. Two distinct measurement approaches were explored: (1) single measurement and (2) multiple measurements performed on each electrode, in which their detection sensitivity and selectivity were compared and discussed. Furthermore, we also investigated the effects of electrode aging and sterilization to access the remaining electrocatalytic activity of Pt/Ni nanocatalysts.

## Materials and methods

### Fabrication of laser-induced carbon nanofibers

**Scheme 1:**
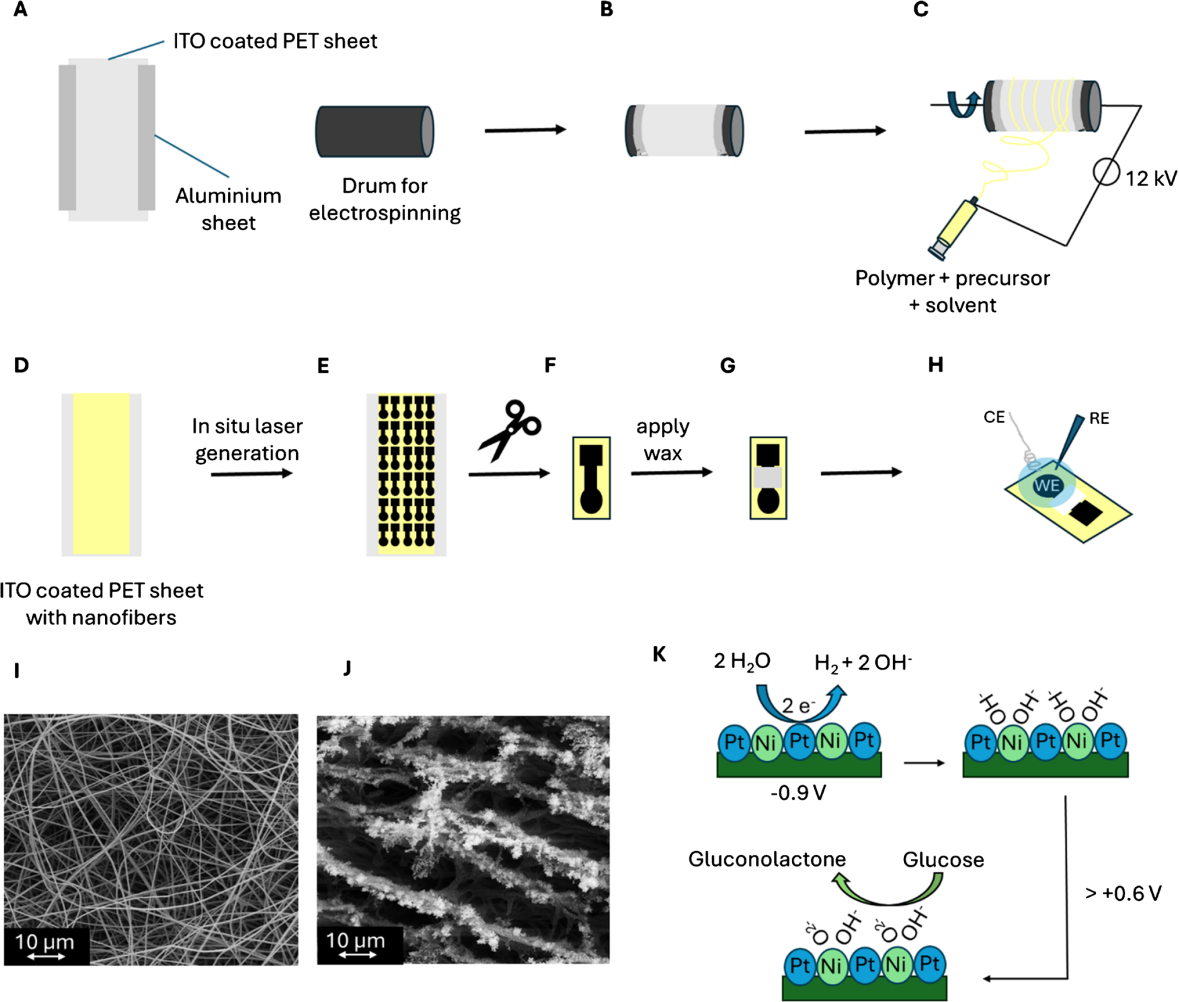
Preparation process and detection mechanism. (**A**) An aluminum sheet is attached to an ITO-coated PET sheet to (**B**) connect it with the rotary drum to enable an electric grounding of the ITO layer. (**C**) Electrospinning nanofibers with a rotary drum (spinning solution: Matrimid®, DMAc, Pt/Ni- acetylacetonate) on the ITO-coated PET sheet. (**D**) Nanofibers collected on the ITO-PET sheet which were further processed by a CO_2_-laser (**E**) to laser induced carbon nanofibers (LCNFs). (**F**) Single electrodes were cut from the sheet with scissors and (**G**) a layer of wax was applied to ensure a defined area of the working electrode. (**H**) The measurement setup with the Pt/Ni-LCNF working electrode (WE), a Pt wire as a counter electrode (CE), and a Ag/AgCl reference electrode (RE). An SEM image (2500 × magnification) of electrospun nanofibers (**I**) and LCNF (**J**). (**K**) The illustration of the detection mechanism containing an electrochemical pretreatment at − 0.9 V to induce a local high pH and in situ generate Ni(OH)_2_ species, followed by a cyclovoltammetry scan. Above + 0.6 V, Ni(OH)_2_ is oxidized to NiOOH, which is capable to oxidize glucose to gluconolactone

For electrospinning, a polymer solution containing 15% (w/v) Matrimid® 5218 (Huntsman Advanced Materials BVBA, Belgium) in N,N-dimethylacetamide (Merck, Germany) was prepared. Additionally, 25% w/w metal salt (based on the dry mass of the polymer) was added, specifically platinum(II)-acetylacetonate (97%, Sigma-Aldrich, Germany) and nickel(II)-acetylacetonate (95%, Sigma-Aldrich, Germany), in various molar ratios (1:0, 3:1, 1:1, 1:3, 0:1). These mixtures were called 100Pt, 75Pt/25 Ni, 50Pt/50 Ni, 25Pt/75 Ni, and 100 Ni, respectively. The resulting solutions were stirred overnight. Electrospinning was performed following a method similar to that in a previous publication [18]. Specifically, a rotary drum collector with a rotation speed of 150 rpm and a 15 cm distance between the needle (20 G) and collector was used. The applied potential during electrospinning ranged between 12 and 13 kV, at a flow rate of the spinning solution of 10 µL/min. As an additional substrate for collecting nanofibers, an indium tin oxide–coated poly(ethylene terephthalate) sheet (ITO/PET) with a sheet resistivity of 60 Ω/sq (1 ft × 1 ft × 5 mil, Sigma-Aldrich, Germany) was employed. The substrate, cut into pieces of 10 × 30 cm, was attached to the collector of the rotary drum, with the non-conductive side touching the collector. The grounding of the conductive side of the substrate was achieved using aluminum foil, which made contact with both the substrate and the rotary drum collector (Scheme 1A–C). The spinning time was set to 3 h, and the temperature ranged between 22 and 28 °C, with humidity maintained between 30 and 50%. After spinning, nanofiber mats (Scheme 1D, I) were dried at room temperature overnight in a fume hood to remove any organic solvent residues.

Laser-induced carbon nanofibers (LCNFs) were generated using a CO_2_ laser (10.6 µm, VLS 2.30, Universal Laser System, Polytech Systeme GmbH, Germany) (Scheme 1E, J). The lasing speed was maintained at 60% (1270 mm∙s^−1^), the image density was set to 1000 DPI, and optimal reproducibility was achieved with a laser power of 2.4 W. The choice of a 2.4 W laser power is quite deliberate to ensure that the laser fully converts the nanofiber layer into laser-induced graphene under this setting. However, the thickness of fiber mats, which can be efficiently controlled via electrospinning time, distance between collector and spinneret, and/or size of collector, is a crucial factor in governing the optimal lasing conditions, i.e., thicker fiber mats require stronger laser power and slower speed than thinner ones. The over- or undercarbonized electrodes typically result in poor electron transfer kinetics which can be obviously seen from resulting electrochemical signals. Nevertheless, measurement of electrical resistance of the as-produced electrodes could be helpful to ensure their reproducibility prior use.

The price per electrode, depending on the metal composition, was estimated in Figure S1A for an electrode size shown in Figure S1B.

### Characterization methods

The TEM analysis was performed at a JEOL NEOARM F200 instrument, equipped with a JEOL EDS detector, at 200 kV beam energy. The samples were prepared by scratching LCNFs off the ITO/PET substrate. The resulting powder was suspended in ethanol and ultrasonicated for 1 min. After waiting for 10 min so that the bigger particles would settle, 10 µL of the supernatant solution was deposited on a TEM grid (carbon film on 400 squares copper grid mesh). The grid was dried at room temperature. No further treatment like plasma cleaning was applied.

For SEM, samples were PdAu-sputtered (2.45 kV, 22 mA, 20 s) before the measurement (SEM, Zeiss/LEO 1530, Germany).

### Electrochemical measurements

As a potentiostat, the MultiPalmSens4® (PalmSens, the Netherlands) was used. All measurements were performed with a 3-electrode system, i.e., LCNF as a working electrode, a Pt-wire as a counter electrode, and an Ag/AgCl electrode as a reference electrode for all electrochemical measurements (Scheme 1H). The geometric size of the LCNF was 0.07 cm^2^, isolated from the contact part of the electrode by candle wax (Scheme 1F, G). All measurements were conducted non-stirred with a 40 µL drop of the solution placed on the LCNF, with the Pt-wire and Ag/AgCl electrode dipping into the drop. All electrodes were used (if not stated otherwise) without any pretreatment on the same day they were laser-scribed (usually 1–6 h after scribing).

Glucose measurements:

To investigate the impact of pH on alloyed LCNFs, NaOH (Sigma-Aldrich, Germany) was added to a 1 × PBS solution (Merck, Germany, initial pH = 7.4, 10 mM phosphate buffer, 2.7 mM KCl, 137 mM NaCl) until reaching pH values of 7.7, 11.8, 12.6, or 12.9. Subsequently, these solutions were used to perform three cyclic voltammetry (CV) scans on untreated, fresh LCNF electrodes (scan range from − 0.1 V to + 0.9 V). Additionally, a fourth CV scan was conducted on the same electrode using the same PBS + NaOH solution containing 10 mM glucose.

For investigating pH modulation, LCNFs in a 1 × PBS solution (pH = 7.4) were employed. Prior to the CV scan, electrochemical pretreatments (20 s at − 0.8 V, 20 s at − 0.9 V, and 40 s at − 0.8 V) were applied directly before the CV scan (ranging from − 0.1 V to + 0.9 V). Notably, a pretreatment at − 0.7 V did not enable glucose detectability.

For the single measurement performed on each electrode (used in the calibration curve and aging study), pristine Pt/Ni-LCNF electrode was immersed in a 1 × PBS solution (pH = 7.4, 10 mM phosphate buffer, 2.7 mM KCl, 137 mM NaCl) containing glucose concentration of interest. Following a pretreatment at − 0.9 V for 20 s, a CV scan was performed from 0.0 V to + 0.8 V (Scheme 1K). Contact angles in the aging study were determined on untreated electrodes. For a pretreatment with Tween to enhance wettability of 20-day-old electrodes, a 0.05 w% Tween in PBS solution was placed on the electrode. After 10 s, the drop was removed and washed 3 times with PBS buffer.

For multiple measurements on the same electrode, the following procedure was sequentially carried out: (1) pretreatment at − 0.9 V for 20 s (unless stated otherwise) in PBS; (2) a CV scan from 0.0 V to + 0.8 V; (3) electrode cleaning by application of − 0.2 V for 60 s (unless stated otherwise); and (4) repeating steps (1) to (3) for five times in the new PBS solution for every CV to establish stable background signal; and (5) repeating steps (1) to (3) again for new analyte solution. The sequential electrochemical measurement steps were computed by using a script in the software MultiTrace 4.5 from PalmSense. The script was constructed in the following sequence:
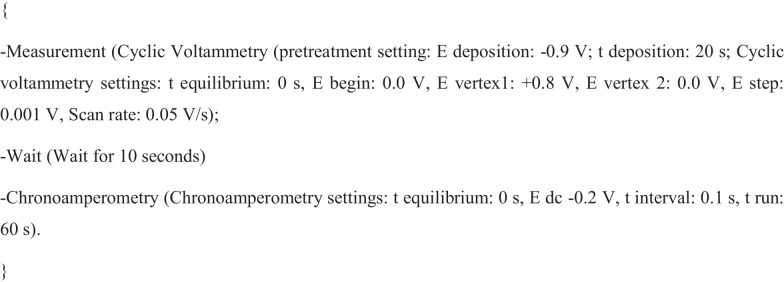


In the recovery study, human serum was bought from Sigma-Aldrich (H4522) and either used pure or diluted by 1:5 in PBS.

## Results and discussion

### Electrode characteristics, and structural and composition analysis

The generation of functional devices containing 2- or 3-electrode systems is feasible when a non-conductive porous substrate, e.g., filter paper, for nanofiber collection as shown in our previous studies [15, 22]. However, the fabrication and manufacturing process are typically more complex and take longer to complete; we therefore fabricated Pt/Ni-LCNFs on ITO/PET and used them as a WE. Furthermore, the use of standard platinum wire and Ag/AgCl as a CE and RE, respectively, in our electrochemical cell could ensure the actual effects that come from the WE material under test, which is our primary focus. Nevertheless, once the Pt/Ni-LCNFs have been well investigated for serving as a WE further manufacturing of device with 2- or 3-electrode system solely from Pt/Ni-LCNFs would be possible.

The electrical resistance of Pt/Ni- LCNF electrodes was 290 ± 5 Ω·cm (*n* > 5) which indicated favorable reproducibility of the production process. In this work, even the mechanical stability of Pt/Ni-LCNF electrodes has not been tested, our previous study has proven that the microstructure of Ni-LCNF on ITO/PET that was used under stirred condition remained unchanged [17]. Furthermore, we aim to use the electrodes under stagnant condition in point-of-care testing and thus believe the mechanical stability should be sufficient.

**Fig. 1:**
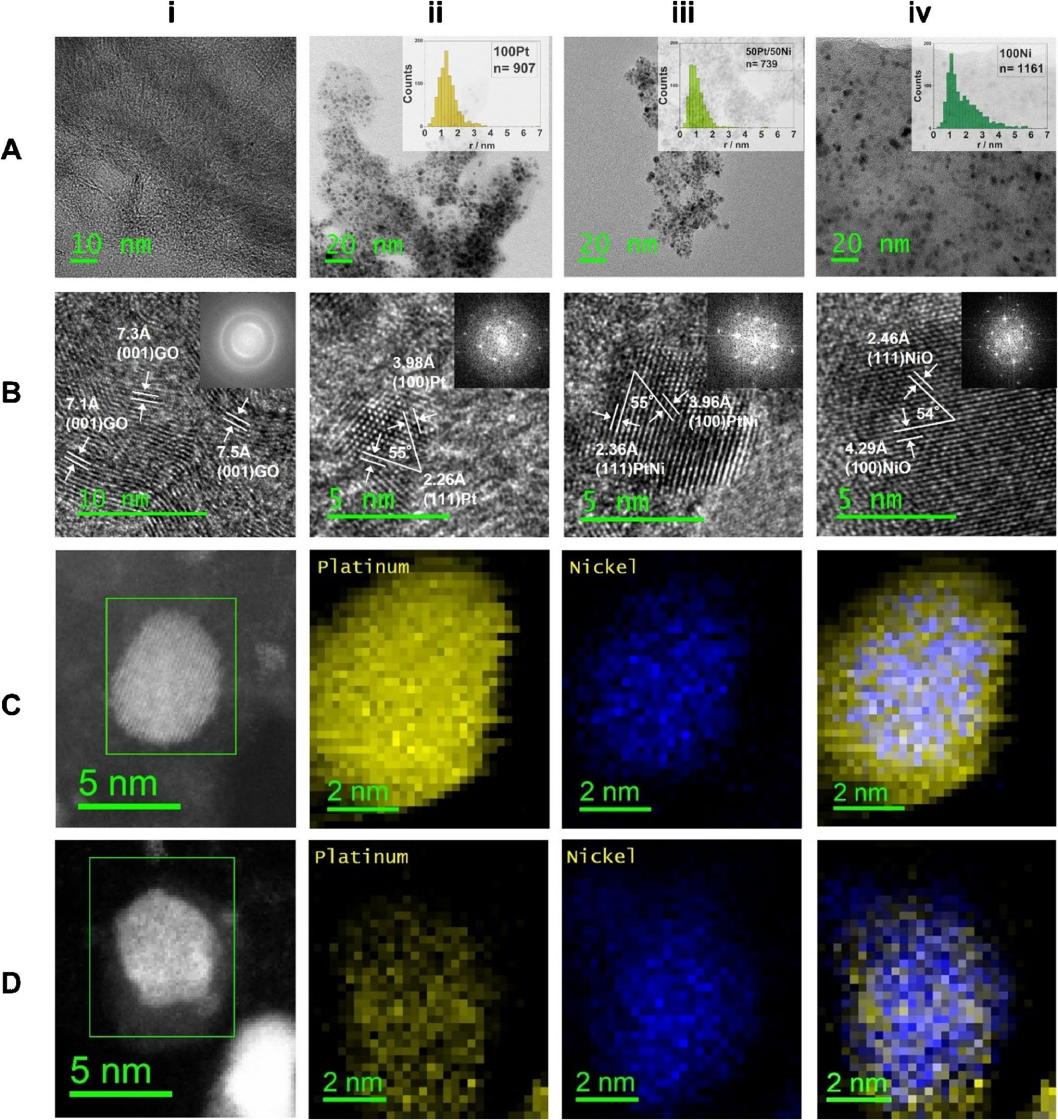
Structural and composition analysis of the laser-generated LCNFs with various Pt and Ni content. **A** An overview of TEM images and the respective size distribution (radius of nanoparticles) of (i) LCNFs, (ii) 100Pt-LCNFs, (iii) 50Pt/50 Ni-LCNFs, and (iv) 100 Ni-LCNFs. **B** The crystalline structure of (i) LCNFs, (ii) 100Pt-LCNFs, (iii) 50Pt/50 Ni-LCNFs, and (iv) 100 Ni-LCNFs revealed by HRTEM. **C** STEM-EDS image showing (i) the mapping area of 50Pt/50 Ni alloy core–shell nanoparticle focused on (ii) Pt, (iii) Ni, and (iv) Pt-Ni overlay. **D** STEM-EDS image showing (i) the mapping area of 50Pt/50 Ni alloy non-core–shell nanoparticle focused on (ii) Pt, (iii) Ni, and (iv) Pt-Ni overlay

We investigated LCNF without metal (Fig. 1Ai), 100Pt-LCNF (Fig. 1Aii), 50Pt/50 Ni-LCNF (Fig. 1Aiii), and 100 Ni-LCNF (Fig. 1Aiv) using transmission electron microscopy (TEM). The average radii of the nanoparticles investigated are as follows: 1.4 ± 0.6 nm for 100Pt, 1.1 ± 0.7 nm for 50Pt/50 Ni, and 2 ± 1 nm for 100 Ni. Figure 1A also provides a detailed size distribution of the radii for each metal composition.

Furthermore, high-resolution TEM (HRTEM) was employed to investigate the crystal structure. Specifically, we examined (i) the graphite-like structure of LCNF and (ii) the metal nanoparticles (Fig. 1B). In Fig. 1Bi, we observed various (001)-d-spacings of the graphite-like carbon matrix, ranging from 0.71 to 0.75 nm. For comparison, pure graphite would exhibit a (001)-d-spacing of 0.672 nm [23]. This result suggests that LCNFs contain several functional oxygen groups, which increase the distance between stacked graphene sheets. An irregular occurrence of these functional groups likely contributes to the varying distances between the sheets. Consequently, we can infer that the carbon matrix is similar to graphene oxide (GO) or reduced graphene oxide (rGO).

Regarding the nanoparticles in 100Pt-LCNF and 50Pt/50 Ni-LCNF (Fig. 1Bii + iii), both exhibit similar (111)- and (100)-d-spacings. These values align with literature data: (111)-d-spacing of 0.226 nm/0.236 nm vs. 0.227 nm for pure Pt [24] and 0.225 nm for PtNi alloy [25]; (100)-d-spacing of 0.398 nm/0.396 nm vs. 0.392 nm for pure Pt [24] and 0.389 nm for PtNi alloy [25]. The measured angle between the (111) and (100) planes of 55° matches perfectly with the theoretical value of 54.7° for a cubic system.

Figure 1Biv shows that 100 Ni particles have a (111)-d-spacing of 0.246 nm and a (100)-d-spacing of 0.429 nm. Comparing these values to XRD measurements in literature [26], we find that they are closer to a NiO lattice rather than a Ni lattice (2θ of (111) equals 37.26° for NiO and 44.50° for pure Ni, resulting in a (111)-d-spacing of 0.24 nm and 0.20 nm, respectively. Furthermore, 2θ of (200) equals 43.29° for NiO and 51.86° for Ni, giving a (100)-d-spacing of 0.42 nm and 0.36 nm, respectively).

Finally, STEM-EDX analysis of different 50Pt/50 Ni particles revealed that out of 10 investigated particles, 70% of them are nanoparticles with a Ni-rich core and a Pt-rich shell as shown in Fig. 1 Ci-iv, and 30% of the investigated nanoparticles have an equal distribution of both elements (Fig. 1 Di-iv). The results suggested that synthesis of nanoparticles during laser scribing tends to be rather random, with a general preference of Ni-atoms to be located inside the particles. However, when the nanoparticles contain higher amounts of Ni, there is a greater likelihood that Ni will be also present on the surface.

The correlation between morphological structures and the metal compositions of Pt/Ni-LCNFs was thoroughly characterized using scanning electron microscopy (SEM) in a previous work [18]. Thus, we only provided the exemplary SEM images of 50Pt/50 Ni-LCNFs in the current study (Figure S1C). The distorted fibrous structure can be expected due to harsh conditions of the single laser exposure process. However, the resulting edges and defects are likely to be beneficial as they may serve as active sites and enhance the accessibility of analytes to the embedded nanocatalysts. For further detail on the microstructures of LCNFs with other metal combinations, the authors guided readers to our previous study [18].

In the previous study, we also performed SEM–EDX analysis to reveal global information on the distribution of nanoparticles over the chosen electrode area and the percentage of Pt, Ni, O, and C found within the Pt and/or Ni-LCNFs. However, as the surface chemistry of Pt and/or Ni-LCNFs play a pivotal role in electrocatalytic reactions of enzyme-free sensors, the SEM–EDX data in the previous study are insufficient. Therefore, we want to further explore these aspects in more detail using X-ray photoelectron spectroscopy (XPS). An in-depth interpretation of XPS data was given in Figures S2 and S3 and revealed that the kind of metal salt added to the spinning solution, e.g., Pt(II)- or Ni(II)-acetylacetonate, has a great impact on the amount of oxygenated functional groups of the LCNFs. Furthermore, XPS data confirmed the formation of a Pt-Ni-alloy when both metal salts were mixed (Figure S4).

Small-angle X-ray scattering (SAXS) is a useful technique for assessing structural information of carbon nanomaterials [27]. To our knowledge, SAXS has never been used for characterization of laser-induced carbon nanomaterials and their hybrids. SAXS revealed that increasing metal content (more pronounced for Ni than for Pt) leads to a greater degree of uniform stacking of rGO sheets in which the detailed discussion is given in Figures S5-S7. The observations were explained by the respective heat conduction capability of both metals.

### Pt/Ni-LCNFs for glucose detection—single measurement per electrode

Originally, we aimed to introduce Pt and Ni into LCNFs to enable the detection of glucose under physiological pH as previously reported by other studies [6, 28]. However, the laser-generated Pt/Ni embedded LCNFs did not provide the result as expected. None of the metal combinations yielded any useful signal for glucose detection at pH 7.7 (Figure S8). This is likely due to the difference in preparation method of Pt/Ni alloy and how Pt/Ni are organized within the carbon nanomaterial matrix, which critically affects the electrocatalytic ability. As shown by Mei et al. [6], PtNi nanoparticles were able to oxidize glucose at physiological pH when attached to multi-walled carbon nanotubes (MWCNTs). However, no catalytic activity was observed when pristine carbon black was used as the matrix.

**Fig. 2:**
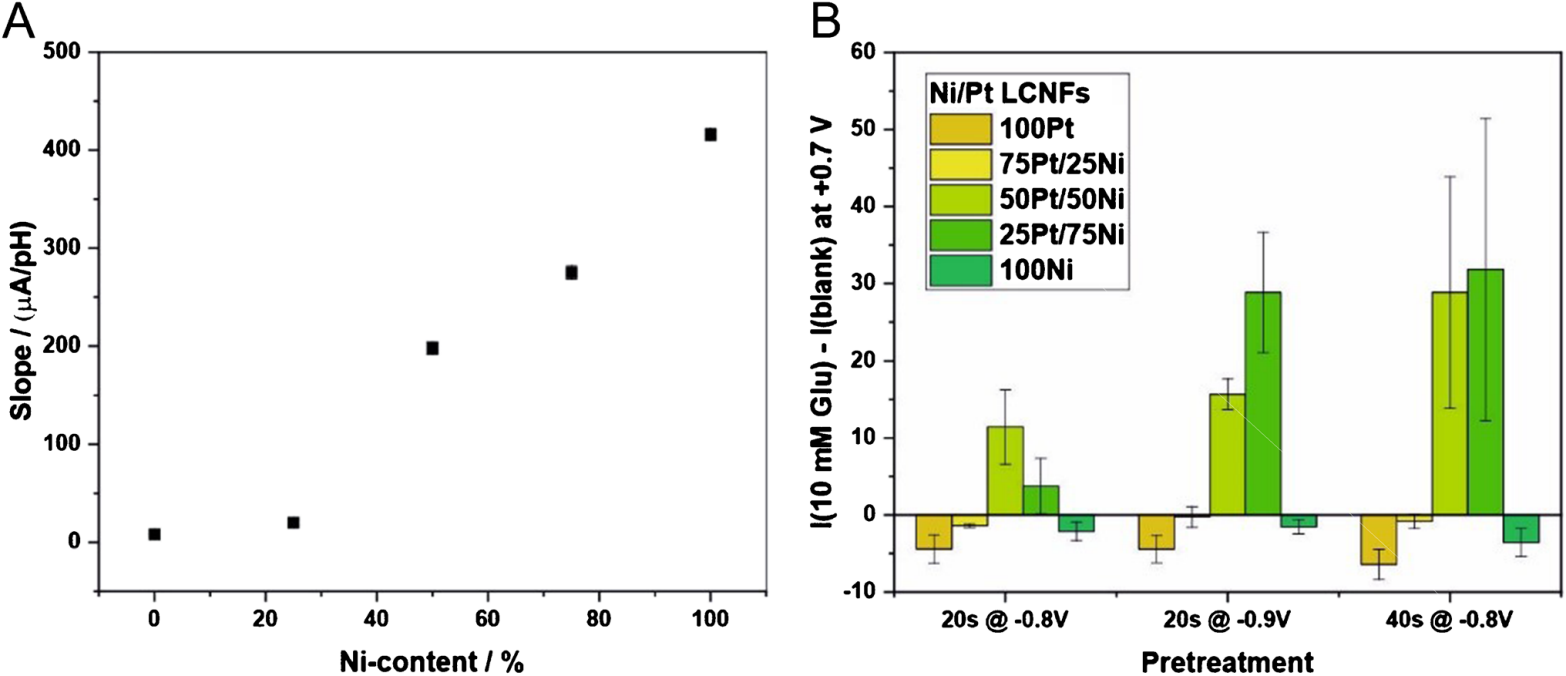
Electrochemical characterizations of electrodes for glucose detection. **A** Impact of Ni content on the sensitivity of glucose considering the anodic currents at + 0.7 V and a pH above 11.5 (Figure S8). **B** Optimization of electrochemical pretreatment conditions for increasing pH locally (*n* = 3)

The electrocatalytic reaction of our LCNF electrodes can be seen only when Ni is present in high contents, and the measurement is performed at basic pHs. This might be explained by the formation of core–shell structure rather than random distribution of Pt and Ni atoms on the nanocatalyst’s surface (see also Fig. 1C) which may hinder the mutual benefit of Pt and Ni in enabling electrocatalytic reaction of glucose at neutral pHs. Figure 2A depicts the linear correlation between detection sensitivity of glucose per pH and Ni content (from 25 to100%) evaluated from Figure S8, suggesting the important role of Ni and OH^−^ for glucose oxidation, as generally known in the following reactions:

1 $${\text{Ni}}^{2+}+ 2\text{ OH}^-\to {\text{Ni}(\text{OH})}_{2}$$

2 $$\text{Ni}{(\text{OH})}_2+\text{ OH}^-\leftrightarrow\text{ NiO}(\text{OH})+\text{ e}^-+{\text{ H}}_2\text{O}$$

3 $$\text{NiO}(\text{OH})+\text{ Glucose}\rightarrow\text{Ni}{(\text{OH})}_2+\text{ Gluconolactone}$$

As it is important to provide OH^−^ to allow the electrocatalytic reaction of glucose to be possible under physiological pH, in situ electrochemically generation of OH^−^ at the Pt vicinity through the water splitting reaction (4 H_2_O + 4e^−^ $\to$ 2 H_2_ + 4 OH^−^) prior to glucose measurement by CV was thus studied. Hereby, the LCNF electrodes containing either Pt or Ni alone did not result in any promising anodic peak from glucose oxidation under the investigated pretreatment conditions (Figure S9). In contrast, the electrodes with Pt/Ni alloy exhibited the expected outcome. Nevertheless, it is necessary to modulate Pt and Ni composition as well as the pretreatment conditions, i.e., voltage and time, to enable efficient in situ generation of OH^−^ and electrocatalytic oxidation of glucose (Fig. 2B). The ratio of Pt and Ni can greatly affect the electrocatalytic oxidation. As the core (Ni)-shell (Pt) structure is formed (Fig. 1C-iv) in this case, a small amount of Ni is unfavorable. This can be clearly supported by the inability of 75Pt/25 Ni-LCNFs to promote electrocatalytic oxidation of glucose even when the electrodes were pretreated prior to glucose measurement (Figure S9). The pretreatment at greater negative voltages and longer times generates higher glucose responses as expected. Here, the 25Pt/75 Ni-LCNFs pretreated at − 0.9 V for 20 s were selected due to their greatest sensitivity, favorable reproducibility, short pretreatment time, and lower cost (less use of Pt). It should be noted that increasing the voltage of pretreatment, e.g., up to − 1 V, adversely resulted in poorer signal reproducibility. Some other previous studies have employed much higher voltages: − 1.5 V for OH^−^ generation and + 1.0 V for 2 s for electrode cleaning [20], and multiple potential steps including − 2.0 V for 20 s to provide the alkaline condition, 0.2 V for 5 s to oxidize glucose, and 1.0 V for 2 s to clean the electrode surface [19]. The lower potential used in our study underscores the efficiency of the bimetallic catalysts and allows the use of more affordable potentiostats that are not capable of applying high potentials, further reducing the overall cost of the setup.

**Fig. 3:**
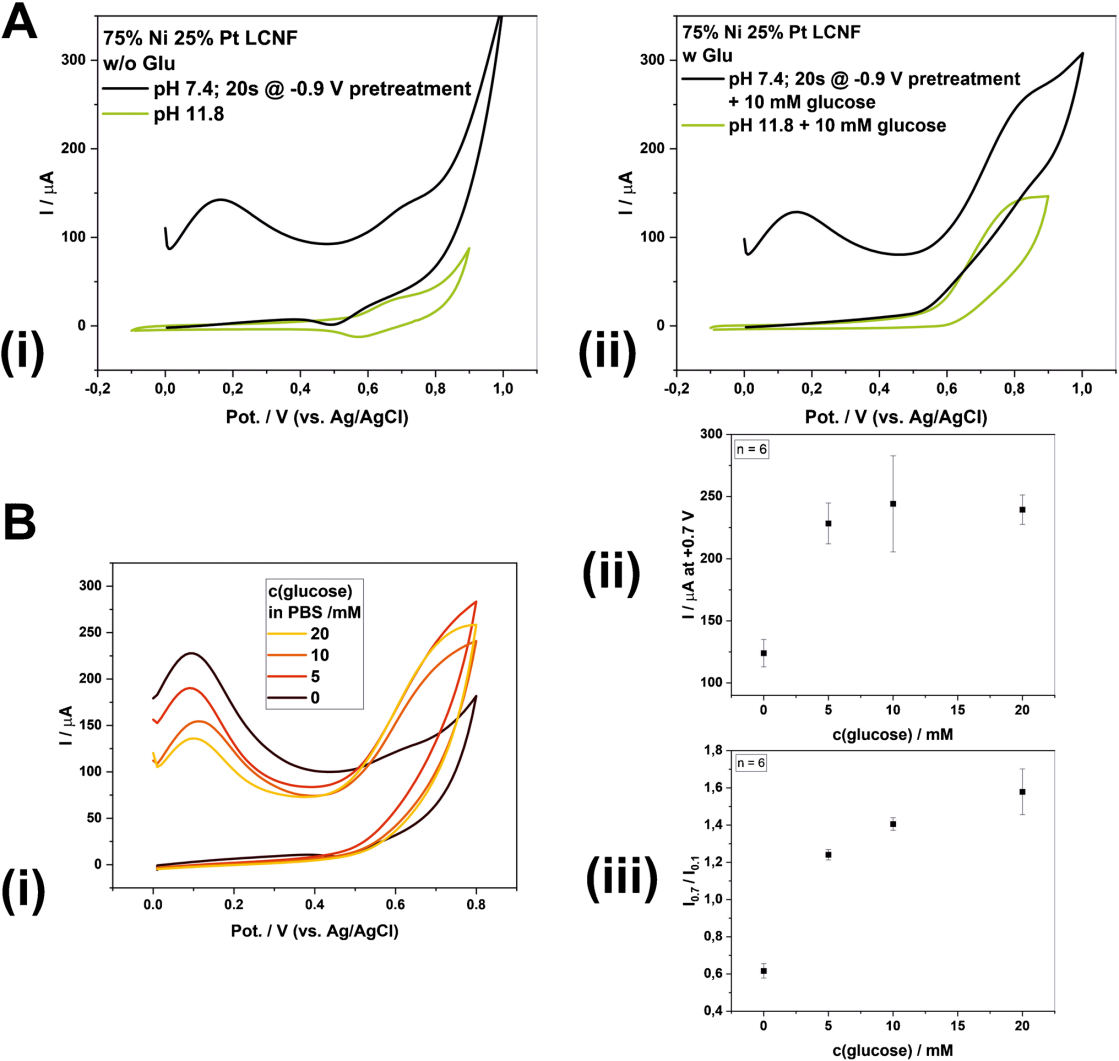
Electrochemical detection of glucose using in situ generation of OH^−^ strategy prior to CV measurement. **A** Proof-of-principle of electrocatalytic reaction (i) without and (ii) with glucose characterized by CV.** B** (i) Exemplary CVs at various concentrations of glucose using pretreatment strategy (− 0.9 V for 20 s), and (ii and iii) improving reliability of data acquisition (*n* = 6). The CV measurement was conducted using a 50 mVs^−1^ scan rate

In order to prove whether the glucose properly undergoes electrocatalytic reaction as commonly achieved from basic solution, we performed CVs that compared the signals of the glucose measurements in PBS buffer with adjusted pH of 11.8 and with electrochemically generated OH^−^ prior to the CV measurements (Fig. 3A). The region from + 0.6 V to + 0.9 V, where electrocatalytic reaction of glucose occurs, is still similar to the case of PBS buffer at pH 11.8 (absence of glucose), indicating the formation of Ni(OH)_2_ species which gets oxidized to NiO(OH) (see Figure S8 and reaction 2) [17]. Additionally, the proper increase of anodic current at around + 0.7 V when glucose is present thus confirmed that in situ generation of OH^−^ assisted by Pt allows glucose measurement even if it is originally present at physiological pH. Furthermore, the intensity of anodic current at around + 0.7 V is proportional to glucose concentration (Fig. 3B-ii) but suffers from undesirable reproducibility (5–16% RSD). However, for freshly used electrodes, the pretreatment produces a non-negligible peak at around + 0.15 V, which is absent when performing CV in PBS buffer pH 11.8. Although we are not clear yet about the source of the peak, the intensity of the peak at + 0.15 V correlates with the peak intensity at + 0.7 V and provides additional information on the activity of the individual electrode. With this, normalization of the signals at + 0.7 V with the maximum peaks at around + 0.15 V enabled the generation of greater reproducible signals with variations at only 2–8% RSD when using a new electrode for each measurement (Fig. 3B-iii).

After the in situ electrochemical pretreatment at − 0.9 V, a strong charging current was observed during the anodic scan (Figure A and B-i) which cannot be seen from the CVs obtained from the basic buffer. The species generated from pretreatment were initially present at high concentration and contributed to the high anodic current. Nevertheless, during the cathodic scan, the current tended to reduce and appeared at similar level as observed from that of the basic solution, suggesting the decrease in the amount of the as-generated electrochemical active species over time. Additionally, the accumulation of in situ generated OH^−^ at the Ni surface immediately after treatment probably results in a strong charging current, which does not occur at the end of the measurement. Accordingly, the starting and end points of CV are not merged when using the in situ electrochemical pretreated electrodes.

Furthermore, the effect of electrode aging was investigated and showed the best performance after 2 days (Figure S10-S11). Further aging drastically worsened LOD due to an increasing hydrophobic character of the electrode. However, a short incubation of the electrodes in a 0.05 w% Tween- 20 solution before measurement solved this issue and enhanced the performance even after 20 days. Additionally, disinfection (incubation in 70% ethanol for 10 min) and sterilization (autoclaved at 121 °C for 15 min) of the electrodes did not harm the electrodes (Figure S12).

### Pt/Ni-LCNFs for glucose detection—multiple measurements per electrode

Even though the single measurement approach shown in section “Results and discussion” offers acceptable detection sensitivity, we still aim to further enhance the analytical performance. The poor wettability and microstructures with rough surface topography of pristine Pt/Ni LCNF electrodes could be a limiting factor for detection ability in the single measurement approach described in section “Pt/Ni-LCNFs for glucose detection—single measurement per electrode” (Figure S1C). In this case, the glucose solution may primarily react with the surface, rather than penetrating deeper into the porous structure. As a result, we attempted to measure the glucose solution using the same electrodes over multiple runs, which could improve surface wettability. However, it is crucial to ensure that the signal from each subsequent measurement remains unaffected by the previous one. Initially, we performed multiple measurements of 10 mM glucose on the same electrode, applying a pretreatment at − 0.9 V for 20 s prior to each CV scan. However, the oxidation current significantly decreased over successive scans (Figure S13 A), likely due to electrode fouling from adsorbed reaction products. To address this, we tested an electrochemical cleaning procedure by applying potentials of − 0.2 V, 0.0 V, or + 0.2 V for 60 s after each CV scan, prior to replacing the old drop with a new drop of glucose solution (Figure S13B). We found that a slightly negative potential (− 0.2 V) was optimal, while potentials of 0.0 V and + 0.2 V worsened the reusability of the electrode. Since the reaction product of glucose oxidation is gluconolactone, which further reacts with water in a hydrolysis reaction to gluconic acid [29], we assume that deprotonated gluconic acid interacts with Ni sites in higher oxidation states (Figure S4). Gluconic acid can be removed from the catalyst surface through a slightly negative electrochemical cleaning step. Without such a cleaning step, the harsh pretreatment at − 0.9 V for 20 s might induce further reactions with gluconic acid, rather than simply removing it from the catalyst surface.

**Fig. 4:**
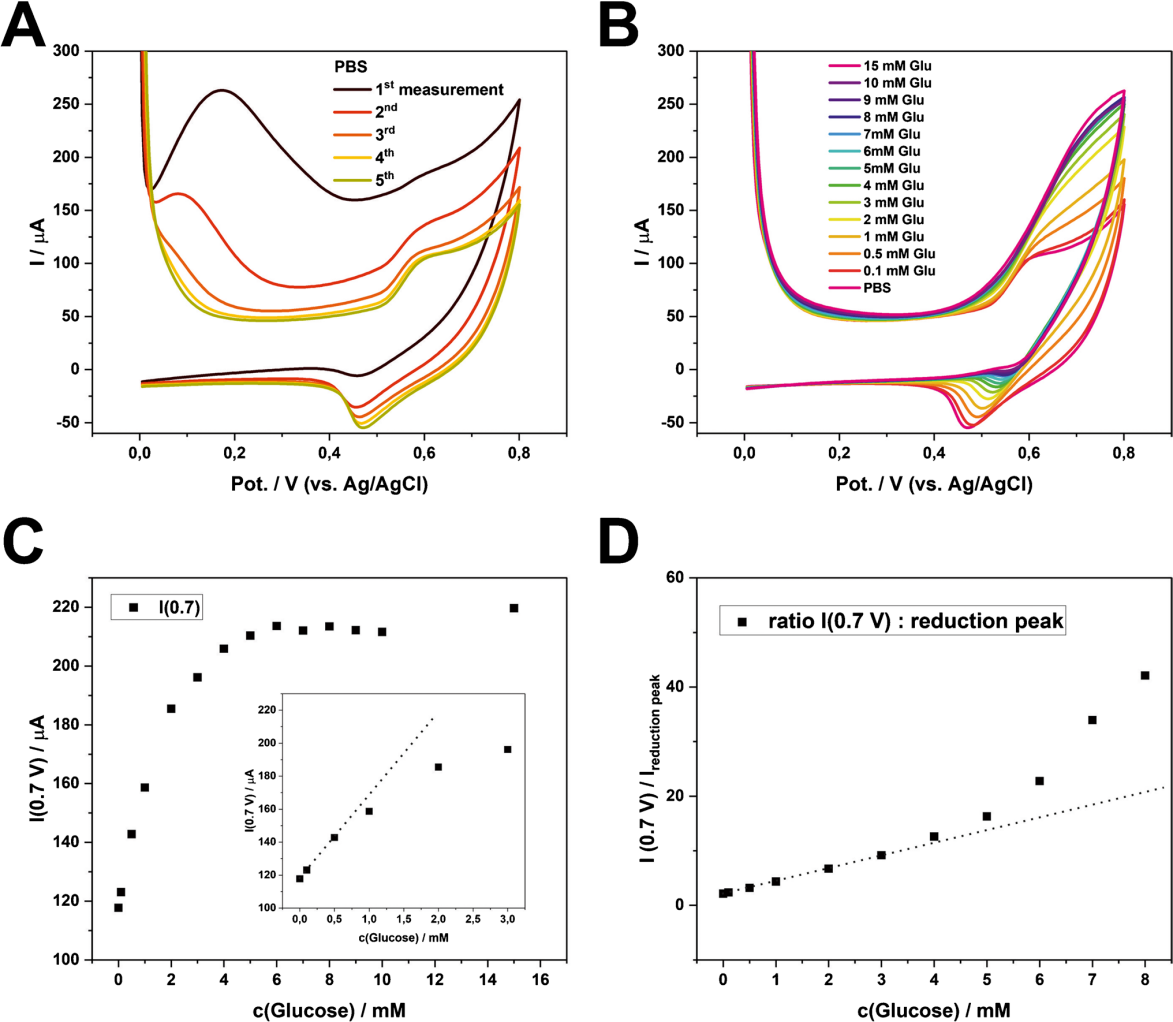
Continuous measurements of glucose on one electrode. A pretreatment of − 0.9 V before each CV measurement was conducted for 20 s. **A** Stabilization of signal and diminishing of background current after 5 CV measurements in PBS. **B** CVs of various glucose concentrations measured consecutively. The CV of PBS is the same CV as the 5 th measurement in (A). **C** Dose–response curve considering the anodic current at + 0.7 V. **D** Dose response curve considering the ratio between the current at + 0.7 V and the reduction peak at + 0.5 V. Since the ratio is a negative value, the absolute value was plotted

In addition, we hypothesized that the detection sensitivity is mainly hindered by the large capacitive background current after OH^−^ generation (Fig. 3). To address this issue, we implemented an electrochemical stabilization procedure to achieve lower background CV signal. Specifically, five CV scans were performed repeatedly in PBS, with a fresh drop introduced for each measurement scan on the same electrode (see section “Electrochemical measurements” for details). Clearly, the stabilization step reduced the capacitive current over the course of the five CV scans. The unknown anodic peaks at around + 0.15 V disappeared, while the peak couples at ca. + 0.7 V and + 0.5 V (corresponding to reaction 2) became more prominent in the later scans (Fig. 4A). We assumed that the repeated CV scans in PBS helped remove edges/defects of LCNFs, thereby exposing more catalytic sites through electrochemical etching.

As expected, the consecutive CV signals show a strong proportional relationship with the introduced glucose concentrations (Fig. 4B). The anodic current at + 0.7 V was taken as the signal response (Fig. 4C), exhibiting Michaelis–Menten behavior with *K*_m_ = 1.3 mM and *V*_max_ = 112 µA. These values were calculated using the equation *I* = $\frac{{\text{V}}_{\text{max}} \bullet \text{c}(\text{Glu})}{{\text{K}}_{\text{m}}+\text{c}(\text{Glu})}$ + *I*(c(Glu) = 0 mM. The *K*_m_ value suggests that at a glucose concentration of 1.3 mM, half of the available catalyst actively undergoes reactions with glucose at a potential of + 0.7 V. The linearity of the curve was observed in the range of 0.1 mM–0.5 mM (*R*^2^ = 0.999; considering the data points at 0 mM, 0.1 mM, and 0.5 mM), resulting in the LOD of 0.06 $\pm$ 0.04 mM (S/N = 3) and a sensitivity of 50 µA/mM.

As an alternative to direct data readout of the anodic peak at + 0.7 V (Fig. 4C), we found that taking the ratio between the anodic current (around + 0.7 V) and the cathodic current (around + 0.5 V) extended the linear range (Fig. 4D). This approach revealed two distinct linear correlations: one between 0.1 and 4 mM (*R*^2^ = 0.994) and another between 5 and 8 mM (*R*^2^ = 0.992), with a LOD of 0.3 ± 0.1 mM (S/N = 3). Compared to Fig. 4C, this data acquisition strategy expanded the linear range by a factor of 8. We hypothesize this following discussion to support why taking the ratio can expand the linear range.

Compared to using the oxidation peak at + 0.7 V for ratiometric data analysis, we understand the meaning of the reduction peak (at around + 0.5 V), which corresponds to the reduction of the catalyst that did not react with glucose:

$$\text{NiO}(\text{OH})+{\text{ H}}_2\text{O }+\text{ e}^-\rightarrow\text{Ni}{(\text{OH})}_2+\text{ OH}^-$$

At concentrations of 0.5 mM and above, sufficient glucose is present to significantly reduce the catalyst during the entire scan. Consequently, even though the catalyst is saturated during the anodic scan, a lower cathodic current corresponding to the reduction peak of the catalyst is additionally observed, further increasing the ratio at higher glucose concentrations. This straightforward approach extends the linear range from 0.1–0.5 mM (Fig. 4C) to 0.1–4 mM (Fig. 4D), as we are no longer only dependent on the maximal reaction rate at + 0.7 V in the anodic scan.

### Selectivity study

Like other enzyme-free electrodes, electroactive interfering species can be problematic, requiring careful investigation. To address this, we examine the effect of interfering species commonly found in blood by adding them to the 1 mM glucose in PBS solution at their relevant clinical concentrations [29], i.e., 0.4 mM uric acid (UA), 125 mM NaCl (nearly double the NaCl concentration in PBS), 0.1 mM ascorbic acid (AA), 0.02 mM cysteine, and a mixture of all of these species.

**Fig. 5:**
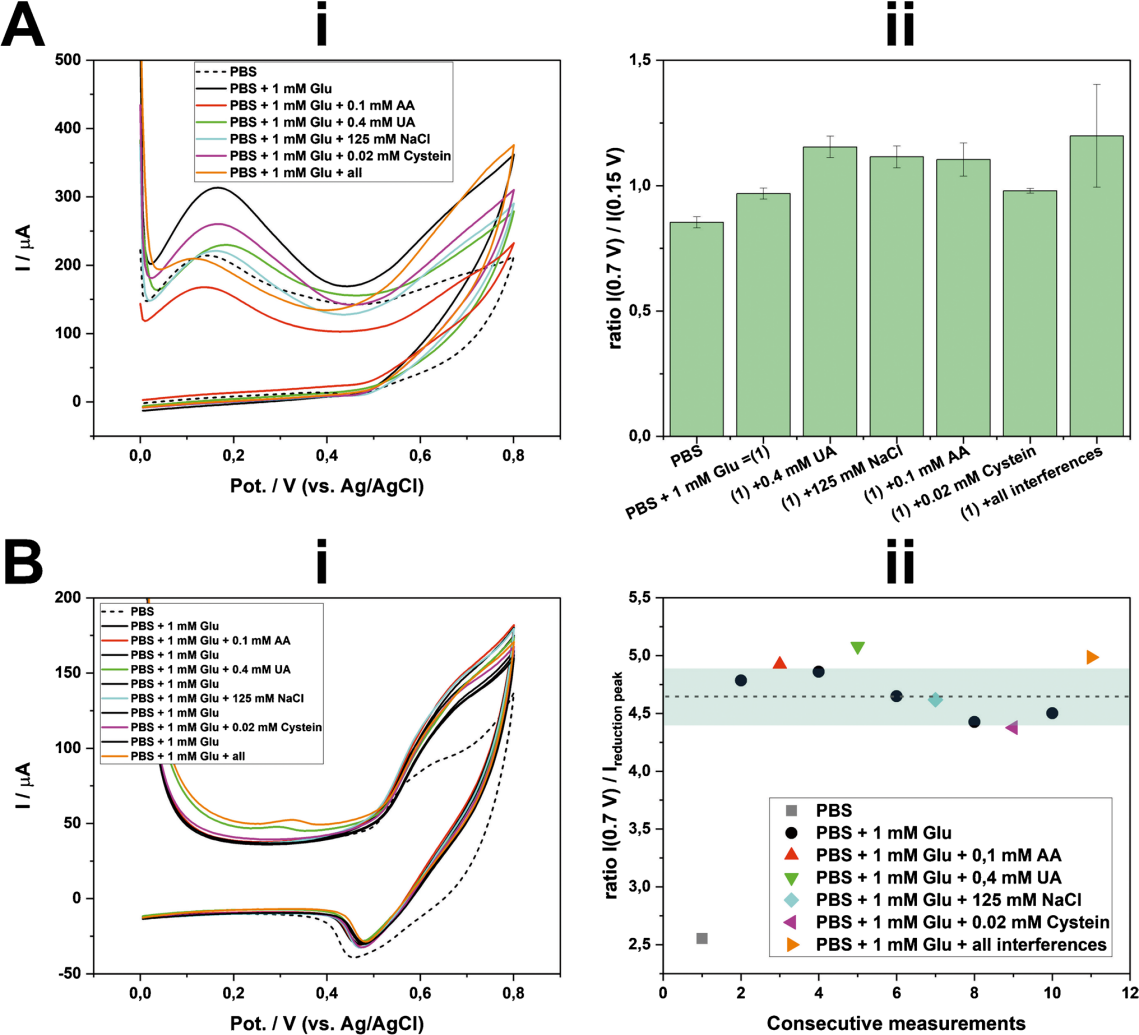
Selectivity study. (**A**) single measurement per electrode approach: (i) the exemplary CVs randomly chosen from all measurements, and (ii) the corresponding self-calibrated signals (*n* ≥ 3). (**B**) multiple measurements per electrode approach: (i) CVs and (ii) the corresponding self-calibrated signals (*n* = 1) where the dash line indicated the mean value of PBS + 1 Glu mM and the highlight indicated its error range

Initially, we assessed the selectivity of these interfering species for the single measurement per electrode approach (see also section “Pt/Ni-LCNFs for glucose detection—single measurement per electrode”). Unfortunately, species such as UA, NaCl, and AA interfered with the glucose measurements (Fig. 5A). This could be due to the high number of edges/defects the pristine Pt/Ni-LCNFs, which may provide favorable site for electroactive interfering species [30]. Additionally, in comparison to our previous study where glucose was measured on Ni-LCNF electrodes in alkaline medium, NaCl at 250 mM showed negligible interference [17]. This suggests that the high background current in the single measurement approach, used to calculate the ratiometric signal, can be easily influenced by interfering species. However, in practical applications involving complex samples like blood, food, and beverage, dilution is commonly performed, which could help mitigate interference effects.

Alternatively, when we conducted the selectivity study using the multiple measurements per electrode approach (see section “Pt/Ni-LCNFs for glucose detection—multiple measurements per electrode”), we observed that this strategy showed high selectivity for glucose, as indicated by the slight deviations in the signal when comparing glucose in PBS with and without interfering species (Fig. 5B). This favorable selectivity could be attributed to the electrochemical etching that occurs during stabilization process (five consecutive CV scans in PBS). This procedure not only eliminates edges and defects where electroactive species such as AA and UA prefer to react, but also exposes more active catalytic sites for glucose, allowing for a more selective reaction. Furthermore, because CV is used, fast reacting species react at lower potentials, reducing their concentrations during the glucose oxidation. The most prominent interfering signal arose from UA, as evidenced by the oxidation peak at around + 0.3 V (Fig. 5Bi). Additionally, consecutive measurements on the same electrode with and without glucose exhibited the high reliability of this approach (Fig. 5B ii). Finally, apart from the superior selectivity, the multiple measurement per electrode approach obviously enables a large discrimination between glucose and background signal (see also Fig. 5 A-ii and B-ii).

### Recovery study

Finally, we assessed the performance of the sensor in human serum using the multiple measurements per electrode approach. The human serum contained 4.8 mM ± 0.1 mM (*n* = 3) glucose, according to measurements made with the commercially available Roche Accu-Chek®. However, we still faced challenges due to the matrix effect when measuring glucose in the human serum without dilution. Specifically, the pure human serum caused a significant reduction in the peaks associated with Ni(OH)_2_ (Figure S14 A), resulting in a self-calibrated signal of 5.4, which corresponds to approximately 1 mM glucose (compared to Fig. 4D). From the shift of the Ni peaks to higher potentials, we concluded that it was not possible to achieve the same local pH increase in undiluted human serum as was achieved with PBS solution. Attempts to increase the pretreatment time (up to 40 s) and apply a more negative potential (up to − 1.8 V) (Figure S14B) did not yield favorable results. We hypothesize that the proteins in human serum predominantly blocked the catalyst on the electrode, thereby hindering the performance of the sensor.

**Fig. 6:**
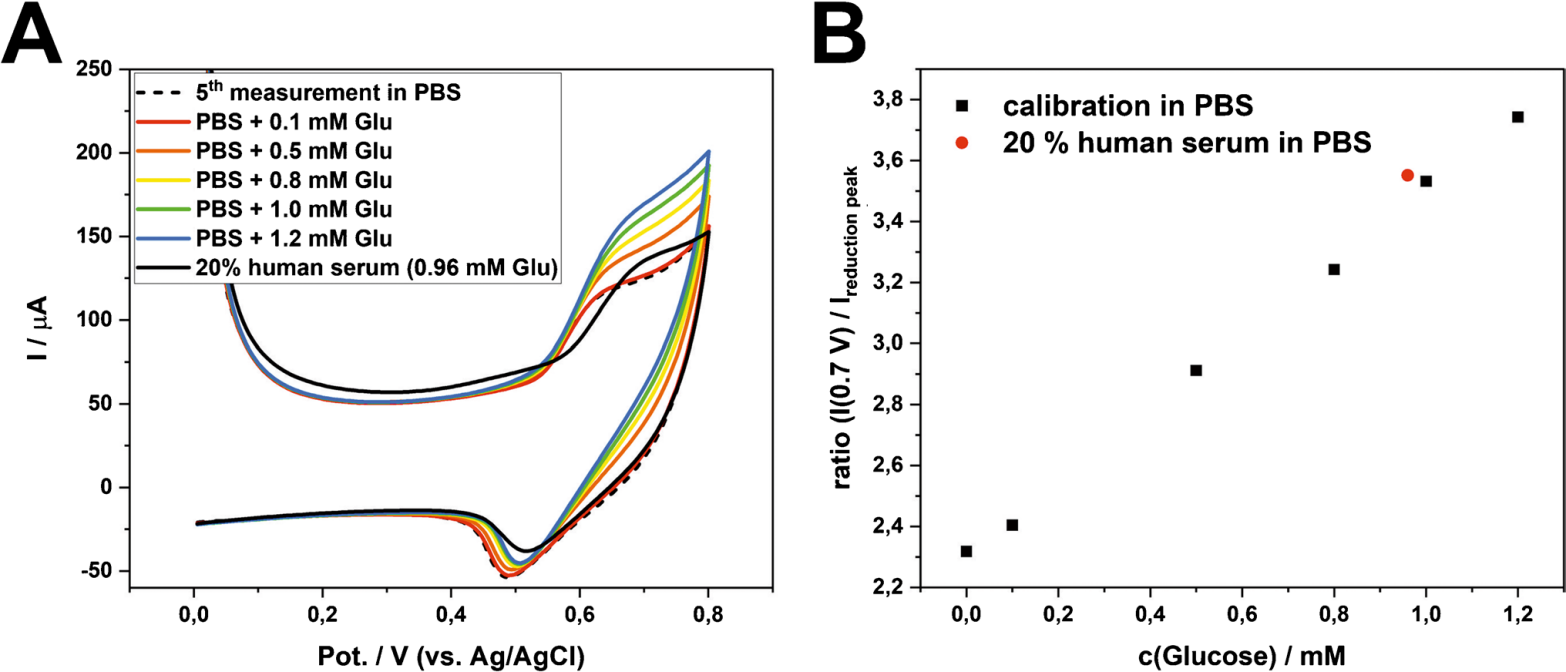
Measurement in 20% human serum. (**A**) CVs of calibration in PBS solution and consecutive measurement in diluted human serum. (**B**) Respective ratios of (A)

Alternatively, we conducted measurements in diluted serum (20% human serum, 80% PBS; resulting glucose concentration, 0.96 mM). We initially recorded a calibration curve (0 mM, 0.1 mM, 0.5 mM, 0.8 mM, 1.0 mM, 1.2 mM glucose in PBS) and then measured either the diluted human serum (20% human serum in PBS; 0.96 mM glucose) or the same solution spiked with 0.25 mM glucose (20% human serum in PBS; 1.21 mM glucose) (Fig. 6). Since good linearity was achieved within the calibration curve (Fig. 6B), we reduced the number of calibration points to a single glucose concentration (Figure S15, electrodes 5, 6) after the stabilization in PBS (5 measurements). Despite the catalyst being blocked by proteins, the ratiometric approach enabled to achieve a good recovery for diluted serum (99 ± 10%), and spiked diluted serum (91 ± 12%) (Table 1). This could be attributed to the self-correction of the CV signal, where both oxidation and reduction currents decreased concomitantly with catalyst blocking. Overall, the recovery for both the spiked and non-spiked measurements was 95 ± 10% (*n* = 6).

**Table 1:** Measurement in undiluted and diluted (20%) human serum. Concentrations are calculated from data shown in Figure S15 (*n* = 3)

Sample	*c* (Roche AccuChek®)	*c* (measured)	Recovery
Undiluted serum	4.8 $\pm$ 0.1 mM	-	-
20% serum 80% PBS	0.96 $\pm$ 0.02 mM^a^	0.95 $\pm$ 0.08 mM	99 $\pm$ 10%
20% serum 80% PBS, spiked	1.21 $\pm$ 0.02 mM^a^	1.1 $\pm$ 0.1 mM	91 $\pm$ 12%

^a^Calculated from measurement in undiluted serum

Lastly, compared to some other reports that performed glucose measurement under physiological pH (Table S1), the Pt/Ni-LCNF electrodes feature low fabrication complexity (as well as cost of material) while their sensing capabilities under stagnant condition are favorable. Especially, in comparison to Au-deposited laser-scribed graphene electrodes reported by Berni et al., even a slightly better detection limit was reported, the detection system required stirring for enhancing mass transport, thus highlighting the advantage of the porous structure offered by Pt/Ni-LCNFs [31]. Nevertheless, as shown in our previous studies, a detection limit can be possibly improved by making an enclosed device where the solution within the microfluidic system can facilitate greater interaction between analyte and electrode surface [15, 22]. Finally, the proximity of Pt and Ni in the form of alloy could promote the use of lower cathodic potential OH^−^ generation when compared to other studies [19, 20, 32].

## Conclusions

We demonstrated the in situ generation of Pt/Ni alloy embedded within carbon nanofibers by CO_2_ laser writing. Through extensive characterization techniques (HR)TEM, STEM-EDS, XPS, and SAXS, we gained valuable insights into the chemistry and structural morphologies of Pt/Ni alloy embedded within carbon nanofibers, shedding light on the effects of the added metals on the materials’ properties. These Pt/Ni alloys embedded in carbon nanofibers were then employed as enzyme-free electrodes for glucose detection, specifically at physiological pH. The beneficial role of Pt/Ni alloy was realized in its ability to efficiently facilitate local OH^−^ generation at Pt sites, which is critical for the electrocatalytic oxidation of glucose by Ni. Consequently, these electrodes offer promising potential for glucose detection under physiological conditions, paving the way for wearable sensor development. The cost-effectiveness and simplicity of electrode fabrication, combined with their favorable electrocatalytic properties and high surface area due to nanoporous structures, straightforward data analysis, and durability, make the laser-generated Pt/Ni carbon nanofibers highly attractive for point-of-care testing applications.

## Supplementary Information

Below is the link to the electronic supplementary material.

Supplementary file1 Price per electrode; Characterization of surface chemistries by XPS and SAXS; Electrocatalytic oxidation of glucose at various pHs and metal compositions; Optimization of pretreatment conditions for electrocatalytic oxidation of glucose present in physiological pH; Effect of electrode ageing and sterilization on electrocatalytic activity for glucose; Reusability of electrodes; Measurement in undiluted and diluted human serum; Comparison of non-enzymatic glucose sensors at physiological pH (DOCX 4.20 MB)

## Data Availability

Data are available from the authors upon request.
