# Supplementary material for: Laser-generated Pt/Ni nanocatalysts-carbon nanofibers enabling self-calibrated enzyme-free glucose detection at physiological pH
Source: Anal Bioanal Chem. 2025 Apr 11;417(15):3337–51. doi: 10.1007/s00216-025-05869-1 (PMC12122661; doi:10.1007/s00216-025-05869-1)
Supplement: Supplementary file 1 — Supplementary file1 Price per electrode; Characterization of surface chemistries by XPS and SAXS; Electrocatalytic oxidation of glucose at various pHs and metal compositions; Optimization of pretreatment conditions for electrocatalytic oxidation of glucose present in physiological pH; Effect of electrode ageing and sterilization on electrocatalytic activity for glucose; Reusability of electrodes; Measurement in undiluted and diluted human serum; Comparison of non-enzymatic glucose sensors at physiological pH (DOCX 4.20 MB) [file 216_2025_5869_MOESM1_ESM.docx]

**Supporting Information**

**Laser generated Pt/Ni nanocatalysts-carbon nanofibers enabling self-calibrated enzyme-free glucose detection at physiological pH**

*Christoph Bruckschlegel,^1^Vivien Fleischmann,^1^ Aladin Ullrich,^2^ Luc Girard,^3^ Pierre Bauduin,^3^ Antje J. Baeumner,^1^ and Nongnoot Wongkaew^1,^**

^1^Institute of Analytical Chemistry, Chemo- and Biosensors

University of Regensburg

Universitaetsstrasse 31, 93053 Regensburg, Germany

^2^Institute of Physics,

University of Augsburg

Universitaetsstrasse 1, 86159 Augsburg, Germany

^3^ICSM, CEA, CNRS, ENSCM,

Univ Montpellier, Marcoule, 30207 Bagnols sur Cèze Cedex, France

*Corresponding author

E-mail: nongnoot.wongkaew@ur.de

**1. Price per electrode**


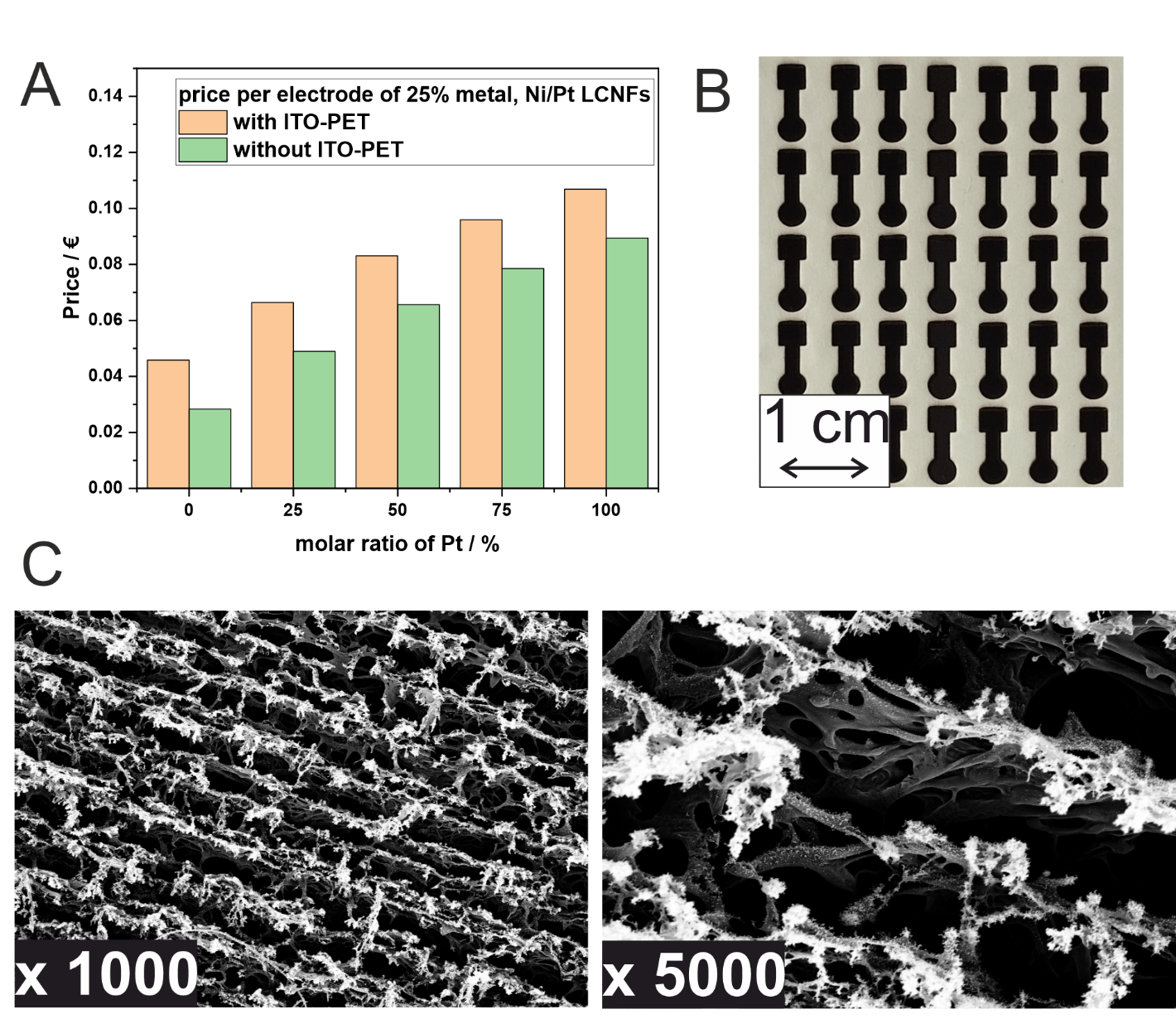


**Figure S1.** (A) Price estimation per electrode when using the scribing pattern shown in (B). (C) SEM pictures of the produced electrode material (LCNF) with a 1000- and 5000-fold and magnification.

Price estimation of the electrode is shown in **Figure S1A** for a scribing pattern shown in **Figure S1B**. We estimated the price per electrode for two different supports (PET/ITO as used in this publication and filter paper, which could be used to further decrease cost). For calculations, a price of 27.2 € per 30 cm x 30 cm ITO/PET sheet, 0.042 € per mL DMAc, 212 € per g Pt(II) acetyleacetonate, 5.74 € per g Ni(II)acetylacetonate, 0.5 € per g Matrimid was used. Furthermore, we estimated that a nanofiber mat (place for 228 electrodes) should consume at the current manufacturing state (stirring overnight, electrospinning for 3h, laser scribing the whole mat) less than 2 kWh, resulting in an additional cost of 0.5 € (assuming a price of 0.25 € per kWh).

**2. Characterization of surface chemistry by XPS and SAXS**

**2.1 Experimental**

The surface composition was monitored by X-ray photoelectron spectroscopy (XPS) on an ESCALAB 250 (Thermo Electron). The X-ray excitation was provided by a monochromatic Al Kα (1486.6 eV) source. Analyzed surface has a 500 µm diameter. The background signal was removed using the Shirley method.[1] The surface atomic concentrations were determined from photoelectron peaks areas using the atomic sensitivity factors reported by Scofield.[2] Binding energies (BE) of all core levels were referred to the C-C of C1s carbon at 284.8eV.

SAXS measurements were performed on a bench built by XENOCS with Mo radiation (λ= 0.071 nm). A q-range from 0.2 to 35 nm^-1^ was recorded on an online scanner detector from MAR Research (diameter: 345 mm). Data analysis was performed with pySAXS. As the sample holder, a self-made powder holder was used (a tube with a diameter of roughly 0.5 cm, closed on both sides with Kapton foil. For background correction, the empty holder with Kapton foil on both sides was subtracted. Silver behenate in a 2 mm quartz capillary was used for scattering vector calibration. For sample preparation, untreated LCNF electrodes were scratched off the ITO/PET substrate and directly filled into the sample holder.

**2.2 Results and discussion**

To reveal the impact of Pt and/or Ni on the graphitic characteristics of LCNFs, the deconvoluted XPS spectrum of C1s orbitals for each type of electrode was divided into four peaks (**Figure S2A**).

**
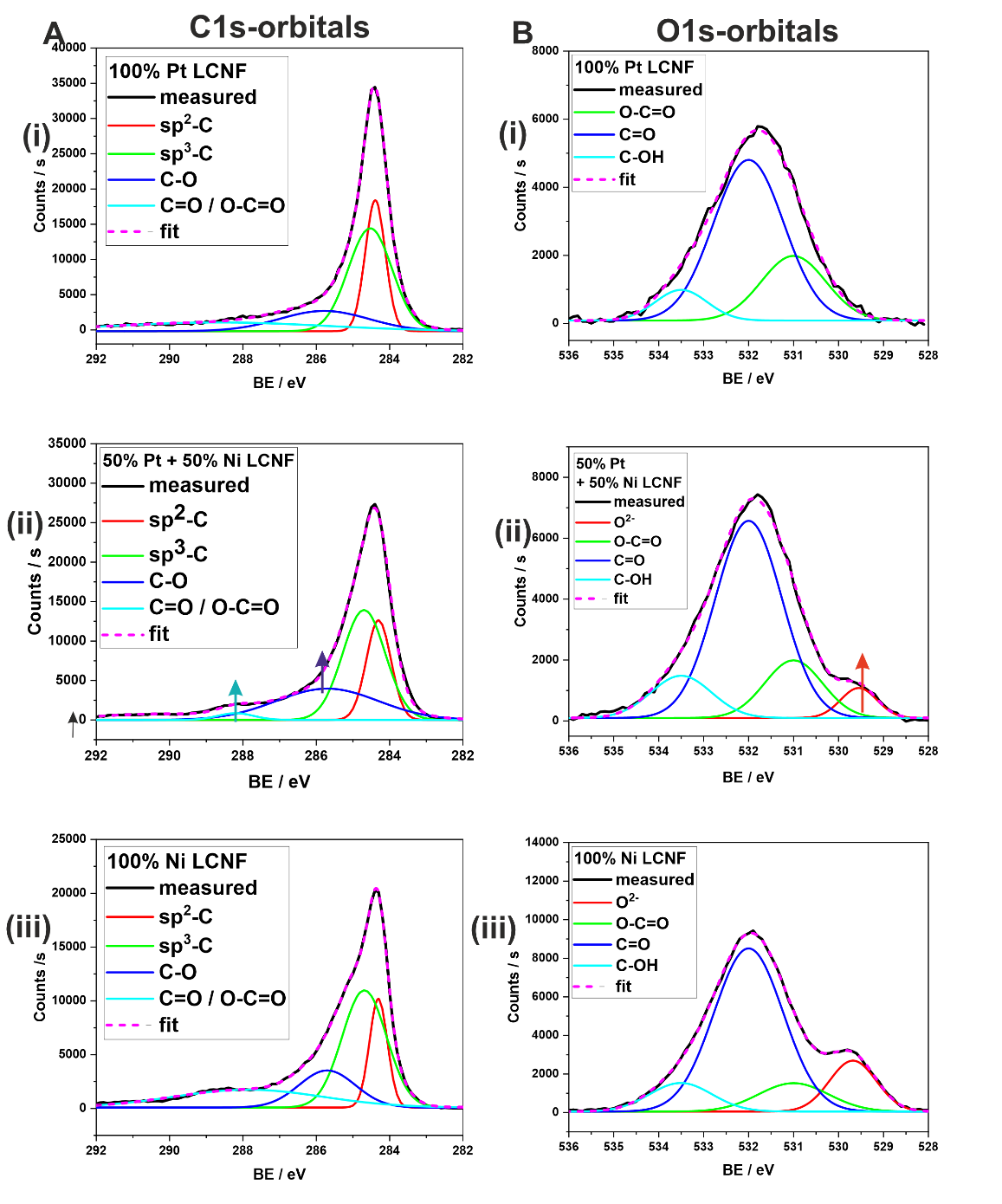
**

**Figure S2.** Deconvoluted XPS spectra with background subtracted of (A) C1S orbitals, and (B) O1s orbitals for (i) 100Pt-LCNFs, (ii) 50Pt/50Ni-LCNFs, and (iii) 100Ni-LCNFs. Blue- and purple-colored arrows indicate an increase of oxygen-containing groups with increasing Ni-content. The red arrow highlights an increasing appearance of O^2-^ with increasing Ni-content, arising from Ni-oxide species.

These peaks were assigned to different carbon environments, i.e., C(sp^2^) at 284.3 eV, C(sp^3^) at 284.7 eV, C-O at 285.7 eV, C=O / O-C=O at 288.3 eV.[3–5] The quantitative evaluation of XPS data shown in **Figure S3A** demonstrated that the 100Pt-LCNFs possessed a greater amount of graphite, indicated by C(sp^2^) peak, than 100Ni-LCNFs. When combining the two metals (50Pt/50Ni-LCNFs), Ni induced a stronger impact than Pt in the formation of sp^2^ hybrid as indicated by the reduction of C(sp^2^) peak by 12% (compared to 100Pt-LCNFs). Considering the presence of edges/defects where oxygenated functional groups are present, 100Ni-LCNFs exhibited O-C=O and C=O contents higher than those of 100Pt-LCNFs. 50Pt/50Ni LCNFs possessed O-C=O and C=O contents similar to those of 100Pt-LCNFs, which may imply that Pt plays a major role in removing these functional groups. This could be due to the lower intrinsic thermal conductivity of Pt that allows better heat localization within PI nanofibers during laser exposure, thus facilitating more complete thermal reduction processes. In contrast Ni, with its higher thermal conductivity, enables faster heat dissipation along the PI nanofibers, leading to less generation of rGO in comparison to 100Pt-LCNFs. For 50Pt/50Ni-LCNFs, Ni seemed to play an essential role in the heat dissipation process as seen from the similar rGO content obtained when compared to 100Ni-LCNFs. Nevertheless, the combination of 50Pt/50Ni promoted the presence of C-O, which is still unclear to us. Overall, it could be concluded that Ni induced the generation of oxygenated groups on LCNFs more than Pt. The data from the C1s orbital (**Figure S3A**) are consistent with those observed for the O1s orbital (**Figure S2B**).


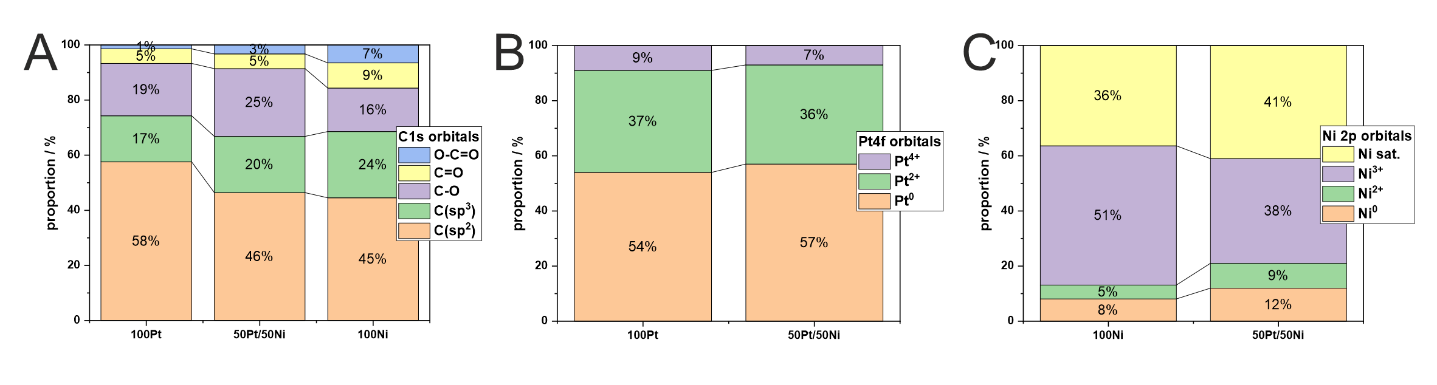


**Figure S3.** XPS analysis of surface chemistry at (A) C1s orbitals, (B) Pt4f orbitals (Pt^0^ and Pt^2+^ and their satellites are combined), and (C) Ni2p orbitals (Ni^3+^ and its satellite are combined).

Considering the XPS spectra of the Pt4f orbital for the 100Pt- and 50Pt/50Ni-LCNFs, the deconvolution of the spectra resulted in five peaks, which corresponded to various oxidation states, i.e., Pt^0^ (at 70.9 and 74.3 eV), Pt^2+^ (at 72.2 and 75.7 eV), and Pt^4+^ (at 77.2 eV) (see also **Figure S4A**).[6]


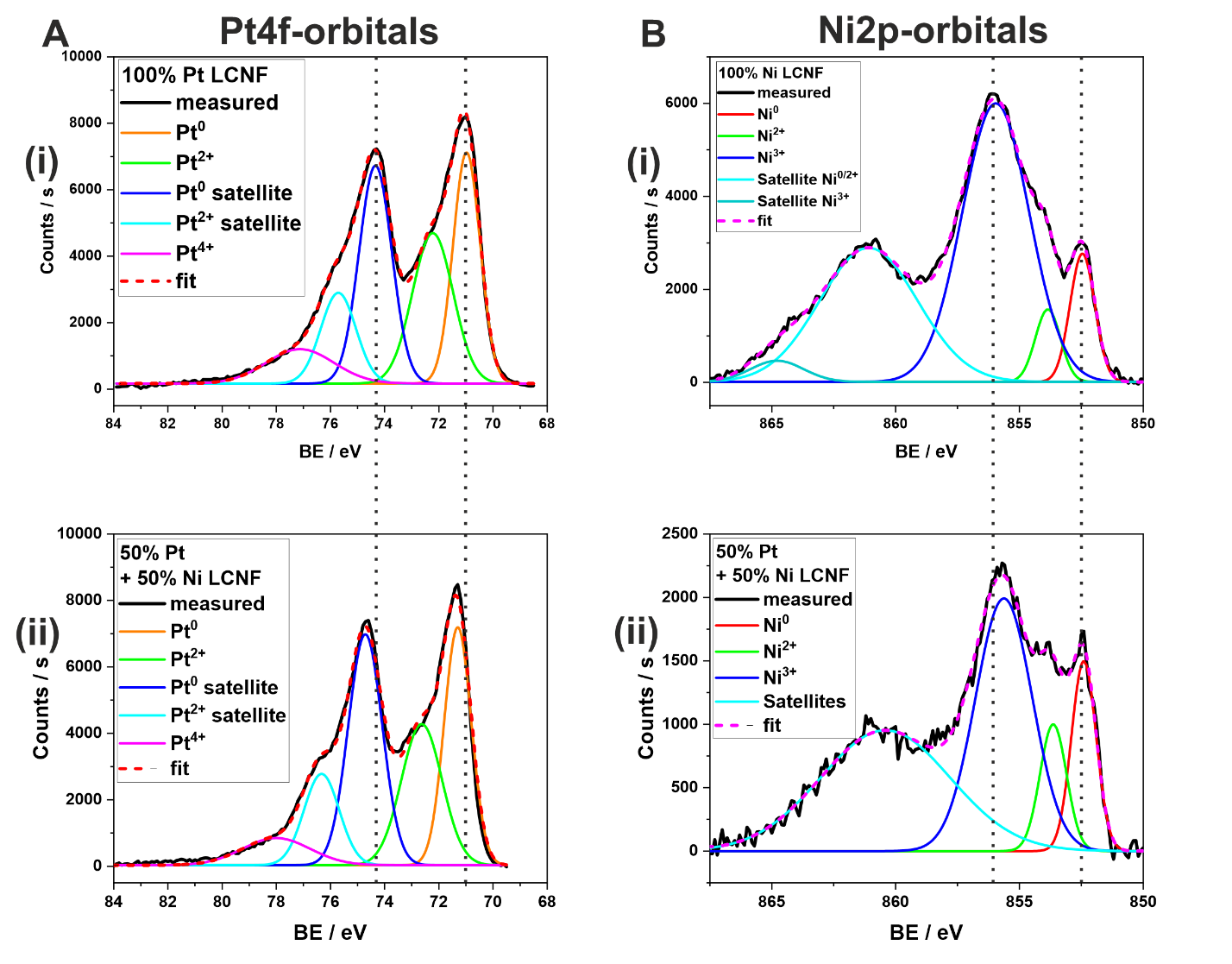


**Figure S4.** Deconvoluted XPS spectra with background subtracted of (A) Pt4f orbitals, and (B) Ni2p orbitals for (i) individual metal either 100Pt-LCNFs or 100Ni-LCNFs, and (ii) 50Pt/50Ni-LCNFs.

Here, Pt^0^ is the dominant species among the others (**Figure S3B**) and has commonly been employed for electrocatalytic reactions in enzyme-free electrochemical sensors. The obtained three valence states of Pt in our study are in accordance with the study in which Pt nanoparticles decorated carbon nanotubes in aqueous solution were generated via femtosecond laser.[7] This work employed potassium hexachloroplatinate (IV) (K_2_PtCl_6_) as the precursor, which is different from ours where platinum(II) acetylacetonate is used. Nevertheless, Liu et al. and co-worker reported the presence of Pt^2+^ and Pt^4+^ when used ultraviolet (355 nm) picosecond laser to convert platinum(II) acetylacetonate mixed with polybenzimidazole (instead of PI) into Pt nanoparticles embedded in graphene films.[8] However, in the work reported by Scroccarello et al.,[9] the major valence state of Pt nanoparticles generated by using CO_2_ laser exposure on the film made from GO and K_2_PtCl_4_ was Pt^0^. These could suggest that the valance states of laser-generated Pt nanoparticles are affected by various factors, i.e., lasing environment including matrices and other compositions where Pt precursor is embedded, type of Pt precursor (counter ion), and type of laser. However, it should be noted that introducing Ni did not significantly alter the content of valence states for Pt (**Figure S3B**). This may be due to its inherent characteristics as a noble metal.

The deconvoluted XPS spectra for Ni2p orbitals of 100Ni- and 50Pt/50Ni-LCNFs displayed in **Figure S4B** revealed the presence of Ni^0^, Ni^2+^, and Ni^3+^ within 100Ni-LCNFs and 50Pt/50Ni-LCNFs. However, unlike Pt shown in **Figure S3B**, **Figure S3C** illustrated the notable difference in the proportions of the valence states when compared between 100Ni-LCNFs and 50Pt/50Ni-LCNFs. Here, Ni^3+^ was the prominent valence state within 100Ni-LCNFs while Ni^0^ and Ni^2+^ were majorly present in 50Pt/50Ni-LCNFs. The decrease of Ni^3+^ and the increase of Ni^0^ and Ni^2+^ in 50Pt/50Ni-LCNFs may be attributed to the intrinsic low heat conduction of Pt that allows more efficient heat localization. In agreement with C1s orbitals, less heat hinders generation of reduced GO (lower rGO:GO ratio), where Ni could serve as a reduction agent. The observation of Ni^0^, Ni^2+^, and Ni^3+^ in our study was consistent with the work reported by Jiménez-Pérez et al. where Pt/Ni electrocatalysts were synthesized via electrodeposition of K_2_PtCl_6_ and Ni(CH_3_CO_2_)_2_ on screen-printed electrodes.[10] It is interesting to point out that the proposed electrodeposition strategy by Jiménez-Pérez et al. also enabled the valence states of Pt^0^, Pt^2+^, and Pt^4+^. The as-developed Pt/Ni porous nanoarchitectures on screen-printed carbon electrodes exhibited superior electrocatalytic activity for H_2_O_2_ detection in the aerosol phase.

Furthermore, according to XPS spectra of Pt^0^ for 50Pt/50Ni-LCNFs (**Figure S4A-ii),** the slight shifts to higher binding energy in comparison to that of 100Pt-LCNFs (**Figure S4A-i**) suggested Pt/Ni alloy formation.[11,12] However, it is worth mentioning that in the previous report,[12] negative shifts of Pt4f were observed in contrast to our case. This might be caused by the difference in the synthesis atmosphere where the Pt/Ni alloy synthesis shown in the literature,[12] proceeded under inert gas while ours was performed under an ambient environment. The evidence of alloy formation was also supported by the shifts of Ni^3+^ and Ni^0^ towards lower binding energies (**Figure S4B)**. To our knowledge, this is the first report elucidating the potential of CO_2_ laser for *in situ* generation of Pt/Ni alloy embedded within carbon nanomaterial matrices, carried out in one-step.[12–14]

A typical SAXS spectrum, which plots the scattered intensity, I(q), against the scattering vector, q, on a logarithmic scale, is shown in **Figure S5**.

**
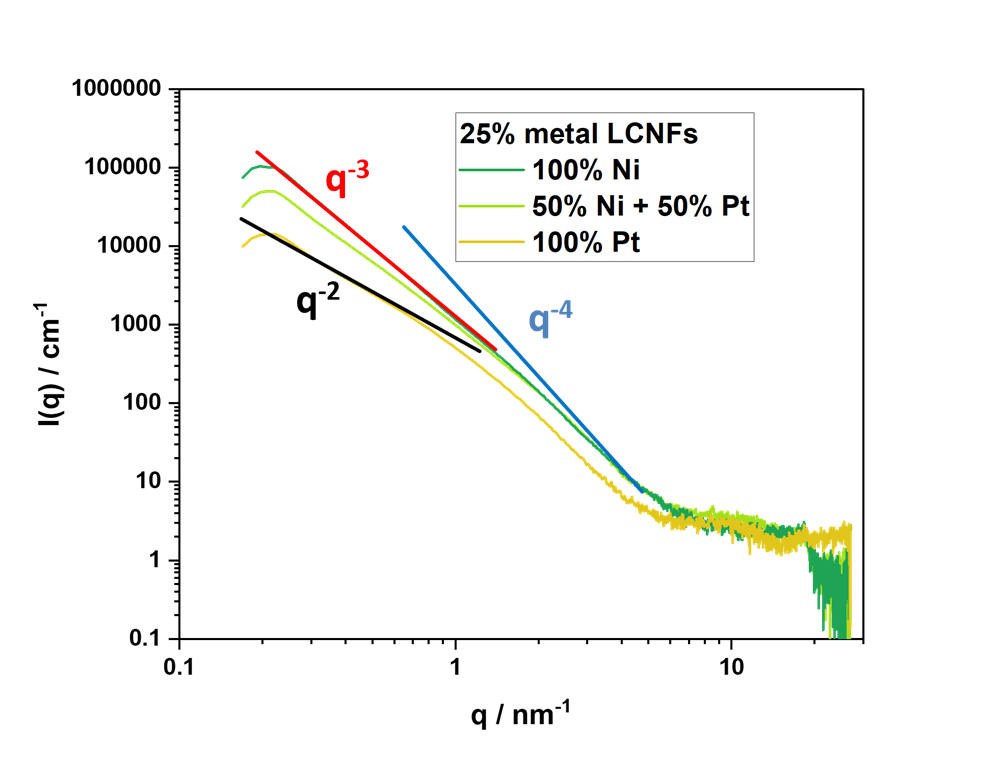
**

**Figure S5.** Full range SAXS spectra and fitted patterns of the as-generated materials.

This figure presents the spectra of 100Pt-LCNFs, 100Ni-LCNFs, and 50Pt/50Ni-LCNFs. Interestingly, only the 100Pt-LCNFs sample exhibits a q^-2^ dependency in the low q region (0.2 to 0.6 nm^-1^), indicating the presence of flat surface structures. These structures, ranging in size from approximately 10 up to at least 30 nm (calculated as (2∙π)/q), reflect the size of reduced graphene oxide (rGO) or graphene oxide (GO) sheets. However, this scattering is largely overshadowed by a q^-3^ dependency, which suggests the presence of undefined structural arrangement of the graphitic sheets within the LCNFs.

To eliminate this overshadowing scattering, we used an I(q) ∙ q^3^ vs. q plot shown in **Figure S6**. **Figure S6** displays the scattering from LCNFs containing various metal compositions and metal concentrations. Interestingly, a broad reflex at 18.7 nm^-1^ emerges with both increasing Ni content (Pt < PtNi < Ni) and increasing overall metal content (15 wt% < 20 wt% < 25 wt%). It is also possible to compare this reflex with X-Ray Diffraction (XRD) data. Here, we converted the q-vector of 18.7 nm^-1^ to its corresponding 2θ angle for copper radiation (λ= 0.154 nm), resulting in 2θ = 26.5˚. According to the literature,[15,16] this angle corresponds to the interlayer distance between two graphene layers, C (002) in crystal structure; a distance of 0.335 nm.


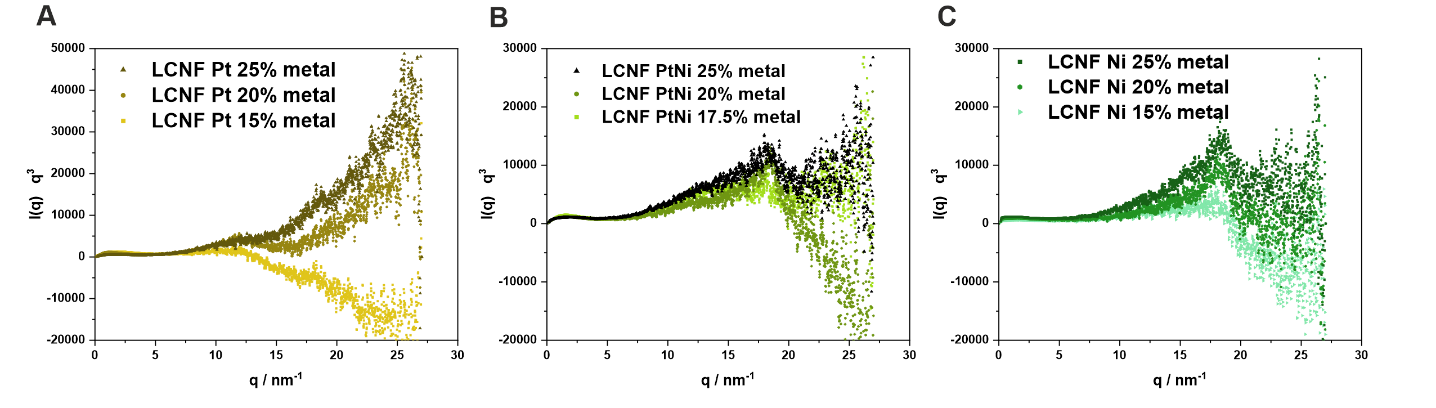


**Figure S6.** I(q) ∙ q3 vs. q plot of 100Pt-LCNFs (A), 50Pt/50Ni-LCNFs (B) and 100Ni-LCNFs with various metal concentrations.

For pure graphite, the reflex at 18.7 nm^-1^ is typically sharp and pronounced due to graphite’s well-defined crystal structure.[15] However, for less defined structures like reduced graphene oxide (rGO), as observed in TEM images in **Figure 1Ai**, a broad reflex similar to the SAXS (I(q) ∙ q^3^ vs. q) plot in **Figure S6B and S6C** is present.[16] Therefore we propose that the more pronounced peak indicates an increasing formation of consistently stacked rGO-like layers. When oxygen-containing groups within the graphite layers are unevenly distributed, the uniform layering structure breaks down, resulting in the disappearance of the reflex at 18.7 nm^-1^. The results presented in **Figure S6** suggest that the type and amount of metal (Ni or Pt) play critical roles in the uniform conversion of Matrimid to rGO. Specifically, Ni appears to be more effective than Pt in such a conversion process. Notably, 100Ni-LCNFs exhibit a greater degree of uniform stacking of rGO, particularly at elevated Ni content, which is not observed in the case of 100Pt-LCNFs. A comparison to HRTEM pictures of rGO structures in 100Ni and 100Pt LCNFs shown in **Figure S7** is in agreement with this observation.


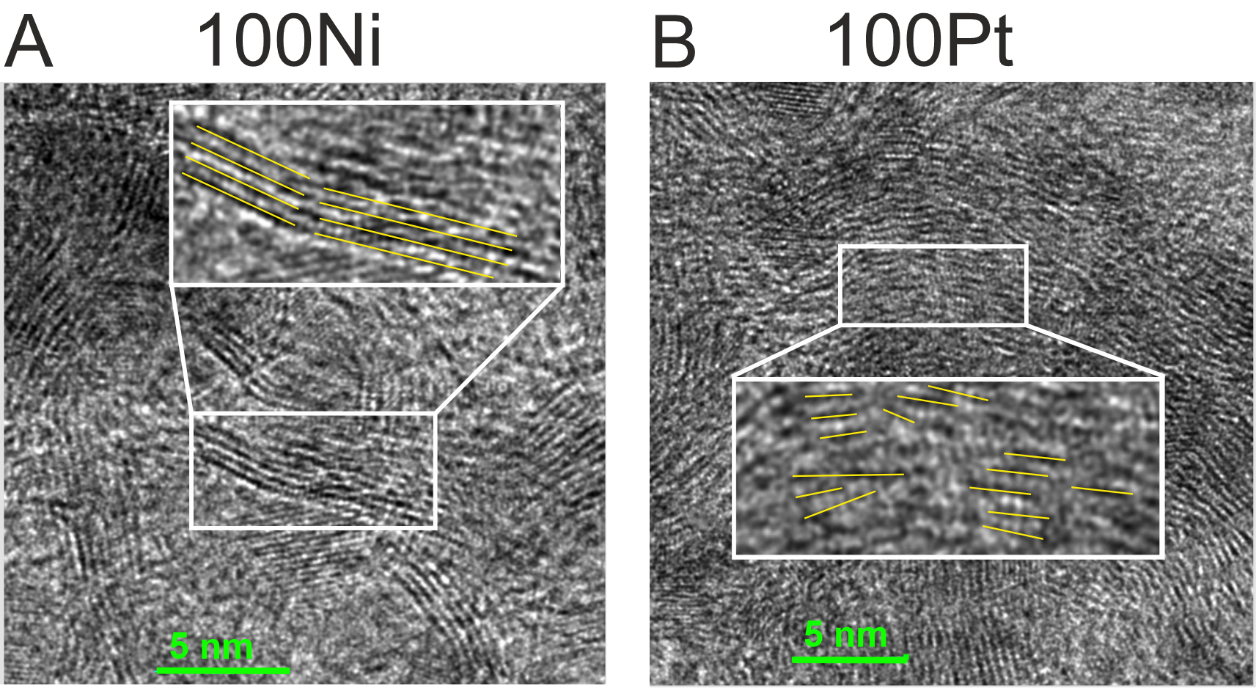


**Figure S7**. HRTEM of (A)100Ni and (B)100Pt LCNFs focusing on rGO structure. An additional zoom highlights the more ordered structure within 100Ni-LCNFs compared to 100Pt-LCNFs, explaining the appearance of the 18.6 nm^-1^ reflex in SAXS spectra of 100Ni-LCNFs

In conclusion, we can propose/hypothesize that the enhanced heat conduction of Ni facilitates fast heat dissipation along the nanofiber precursor, promoting the uniform generation of stacked rGO/GO sheets. In contrast, Pt leads to greater heat localization, resulting in intense local and unevenly distributed heat spots within 100Pt-LCNFs. These heat spots disrupt the uniform stacking, affecting the distribution of oxygenated groups. Hence, SAXS is here a powerful method for elucidating the graphitic structure of LCNFs.

**3. Electrocatalytic oxidation of glucose at various pHs and metal compositions**


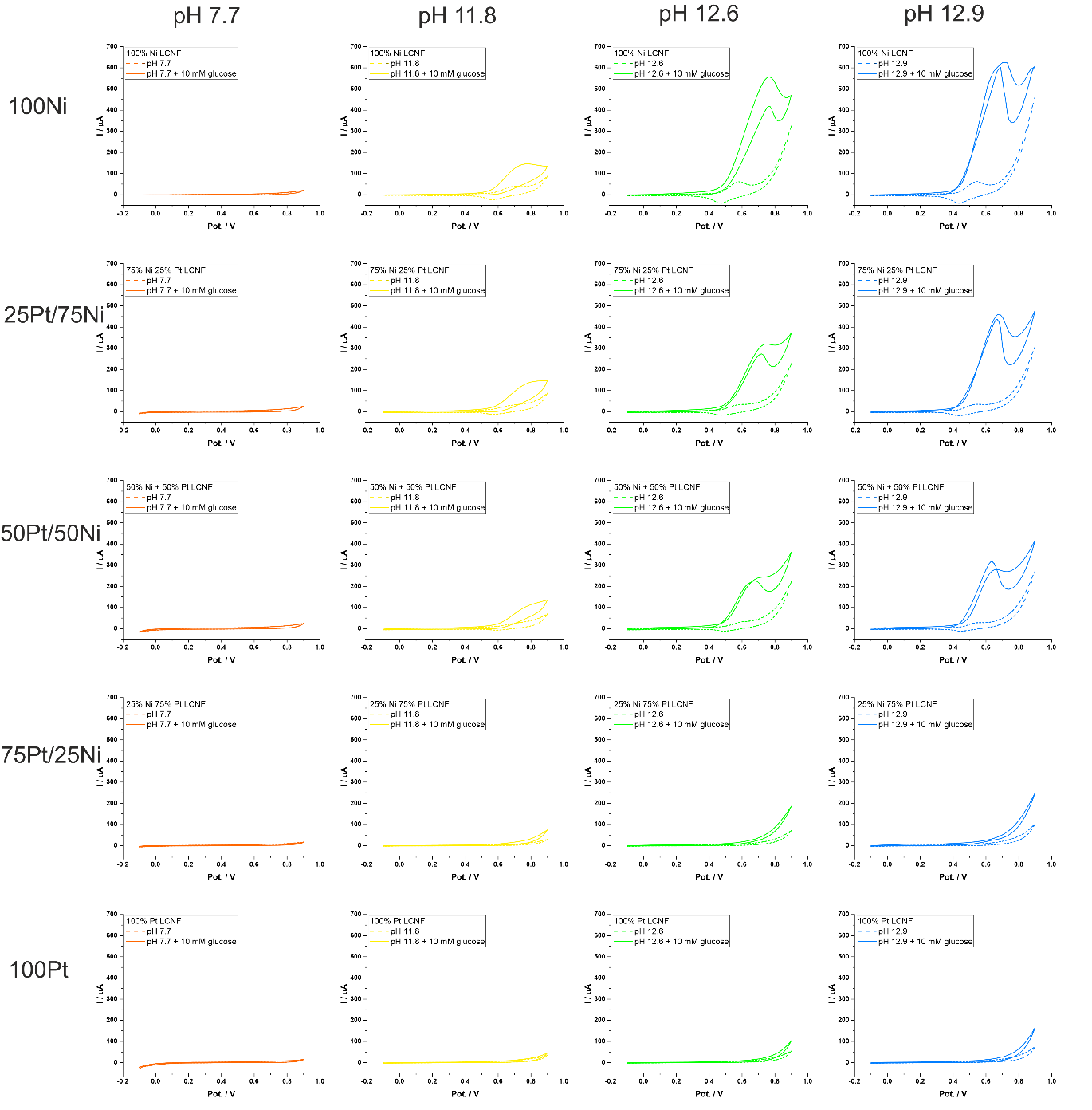


**Figure S8.** Cyclic voltammograms (CVs) of 10 mM glucose (solid line) compared with 0 mM glucose (dash line) from LCNF electrodes contained various metal compositions measured under different pHs. Glucose solutions were prepared in PBS buffer adjusted to the desired pHs by NaOH (1 M). CV was performed using scan rate of 0.05 V/s. Each CV was from the averaged current obtained from individual triplicate measurements.

**4. Optimization of pretreatment conditions for electrocatalytic oxidation of glucose present in physiological pH**


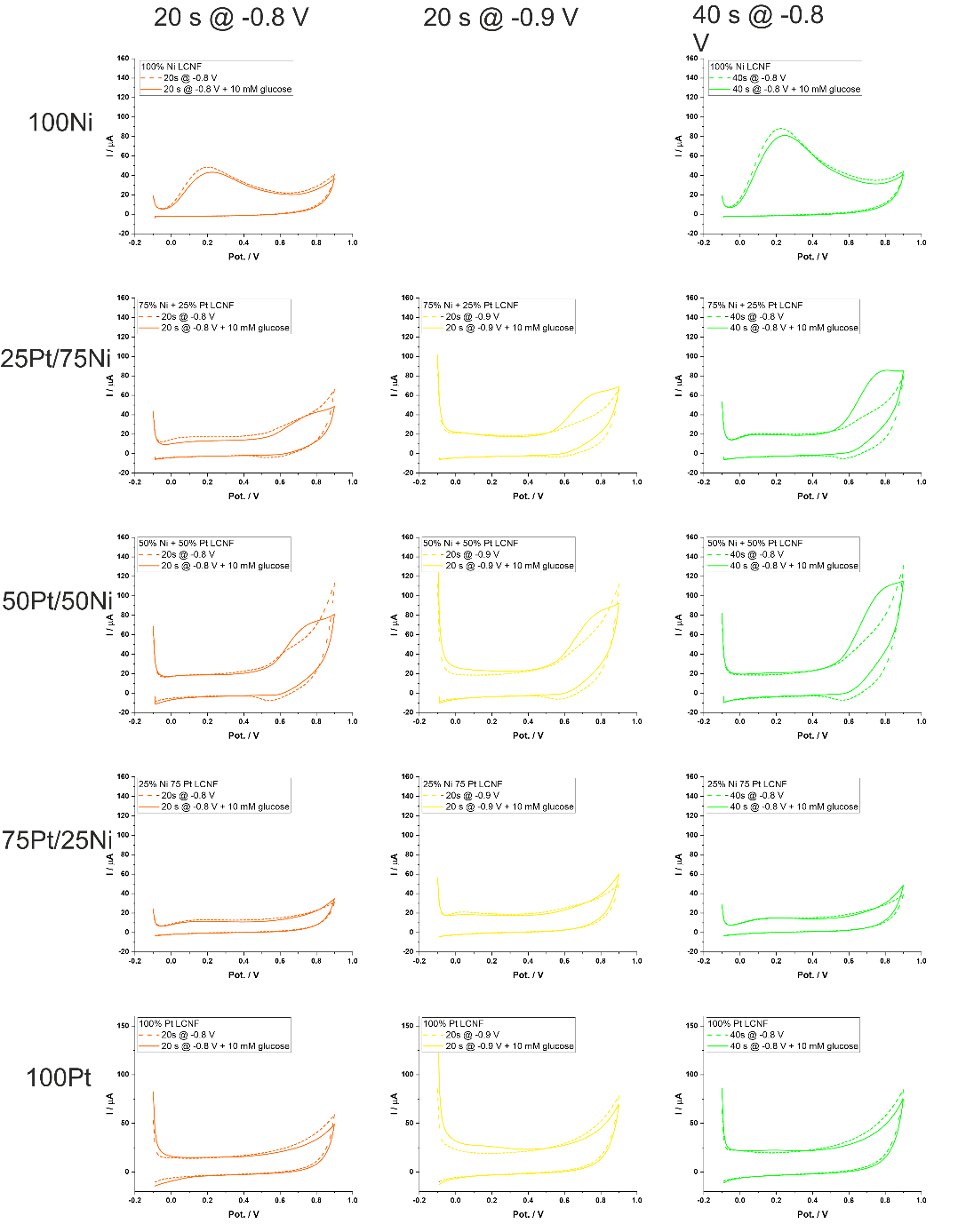


**Figure S9.** CVs of 10 mM glucose (solid line) compared with 0 mM glucose (dash line) from LCNF electrodes contained various metal compositions after pretreatment at various times and applied voltages. Here, the pretreatment condition was applied directly before the respective CV scan, either in the absence or presence of glucose. Glucose solution was prepared in PBS buffer (pH 7.4). The absence of the oxidation peak at around 0.15 V is due to the fact that the electrodes were no fresh electrodes and already performed measurements for Figure S6.

**5. Effect of electrode ageing and sterilization on electrocatalytic activity for glucose**

In general, it is known that electrodes with high surface activity show ageing effects over time when stored under ambient conditions. It was also shown here that storage affected electroanalytical performance and wettability (**Figure S10** and **Figure S11).** In general, high signals were obtained for fresh electrodes resulting though also in high standard deviations and hence worse limit of detection (LOD) than those aged for a few days under ambient conditions. Furthermore, the increase of water contact angle from ca. 100° to ca. 120° well supports the diminishing performance of the sensor with age. Herein, the increase in electrodes’ hydrophobicity prevents homogeneous contact between the analyte solution and the electrode’s surface. This problem can be circumvented by, for example, the addition of a surfactant, e.g., Tween. Here, the dramatic increase in linear correlation and approximately 5-fold improvement in LOD after addition of Tween emphasize the necessity of wettability when using 3D-porous carbon nanofiber electrodes (**Figure S10D to S10F**).


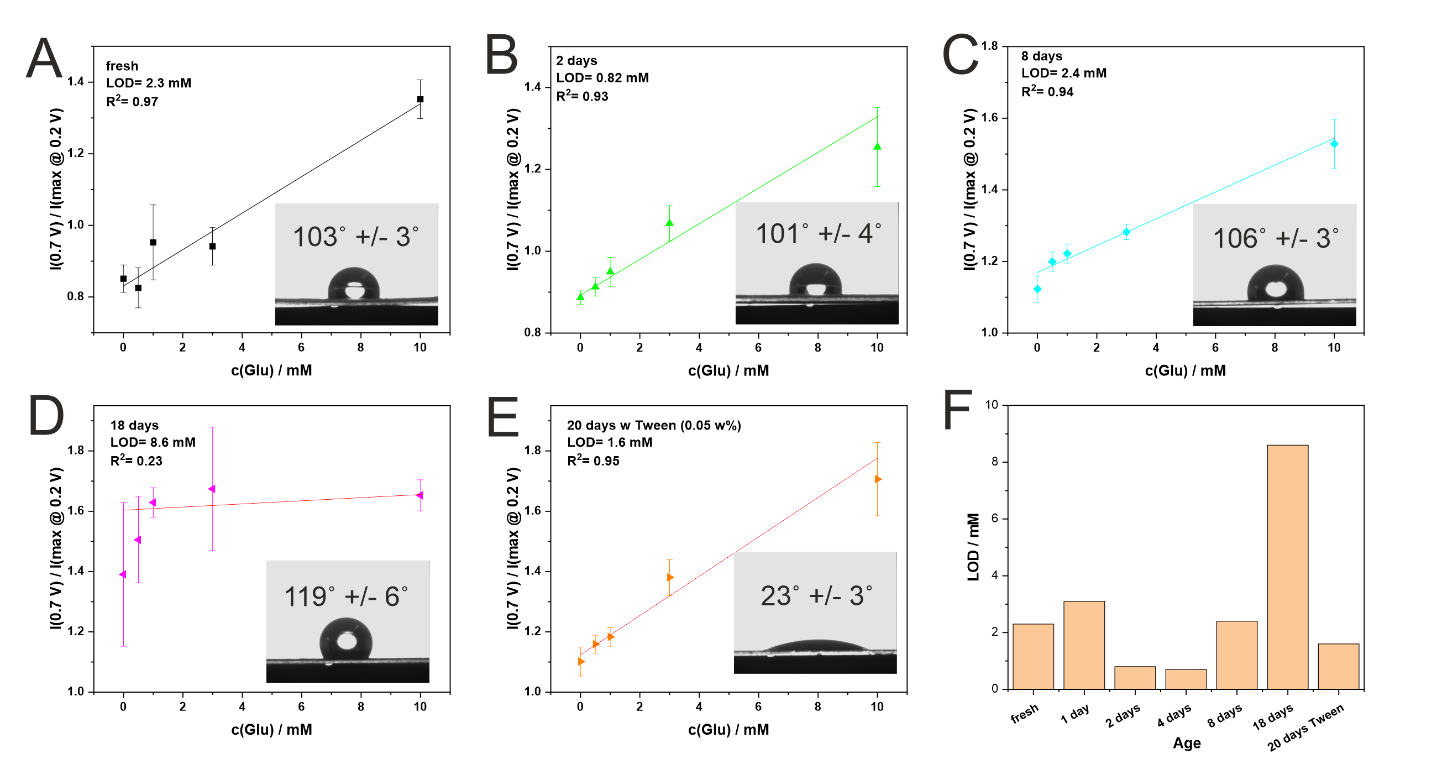


**Figure S10.** Effect of electrode ageing on the detection sensitivity of glucose when electrodes were stored under ambient conditions for 0, 2, 8, and 18 days (A-D, respectively), where the insets display water contact angles for each case. (E) Improvement of analytical performance of 20-day old electrodes by the addition of Tween-20 to the measurement solution (n=5). (F) Summary of LODs from A-E.


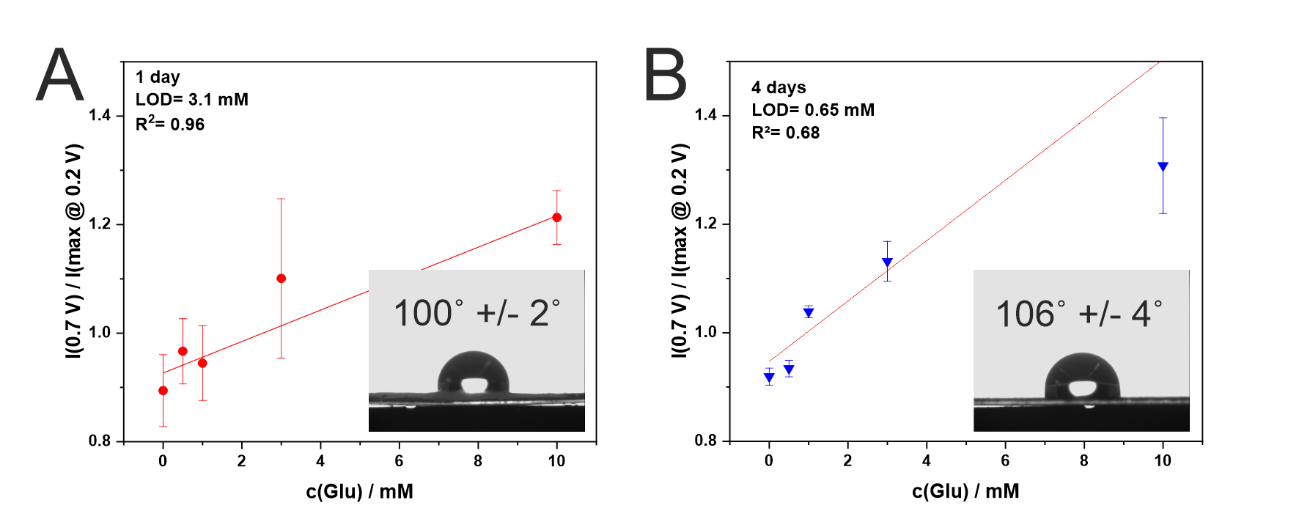


**Figure S11.** Effect of electrode ageing on detection sensitivity of glucose when electrodes were stored under ambient conditions for (A) 1 day, and (B) 4 days.) where the insets display water contact angle for each case. (n = 5)

The best analytical performance using the single measurement per electrode approach was achieved from electrodes aged for two days, resulting in a limit of detection (LOD) of 0.82 mM (S/N = 3), a sensitivity of 0.06 mM^-1^, and a linear range from 0 mM – 10 mM (R^2^ = 0.93).

Bioanalytical applications in general can require the use of electrodes under sterile conditions, which is a major hurdle for enzyme-based biosensors, as the proteins seldomly withstand required sterilization procedures. This opens a unique application field for nanozyme sensors. Therefore, we investigated the effect of sterilization using two common techniques, i.e., heat steam (autoclaved) and 70% alcohol solution. Both sterilization techniques did not result in any obvious change of signal in comparison to non-sterilized electrodes (**Figure S12)**, paving the way for their usability in these areas.


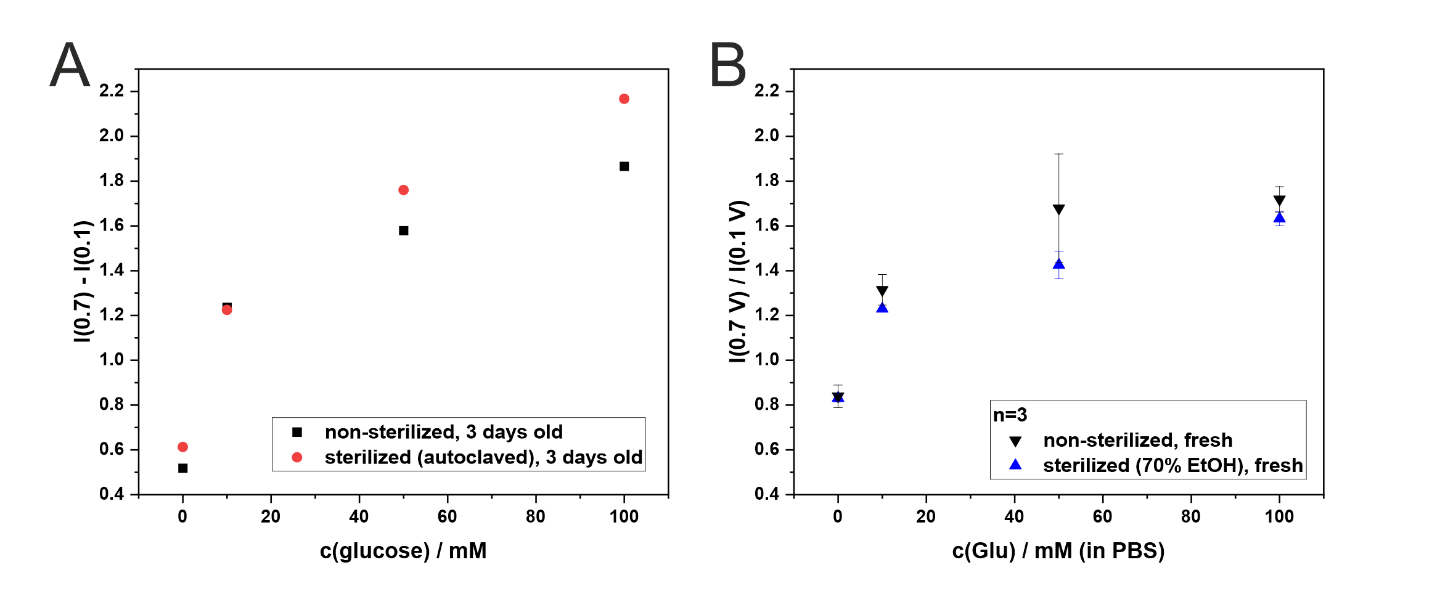


**Figure S12.** Comparison of the glucose signals obtained from electrodes without and with sterilization by (A) autoclaved at 121°C for 15 min, and (B) exposed to 70% ethanol for 10 min. For both conditions, electrodes were dried prior to the measurements.

**6. Reusability of electrodes**

**
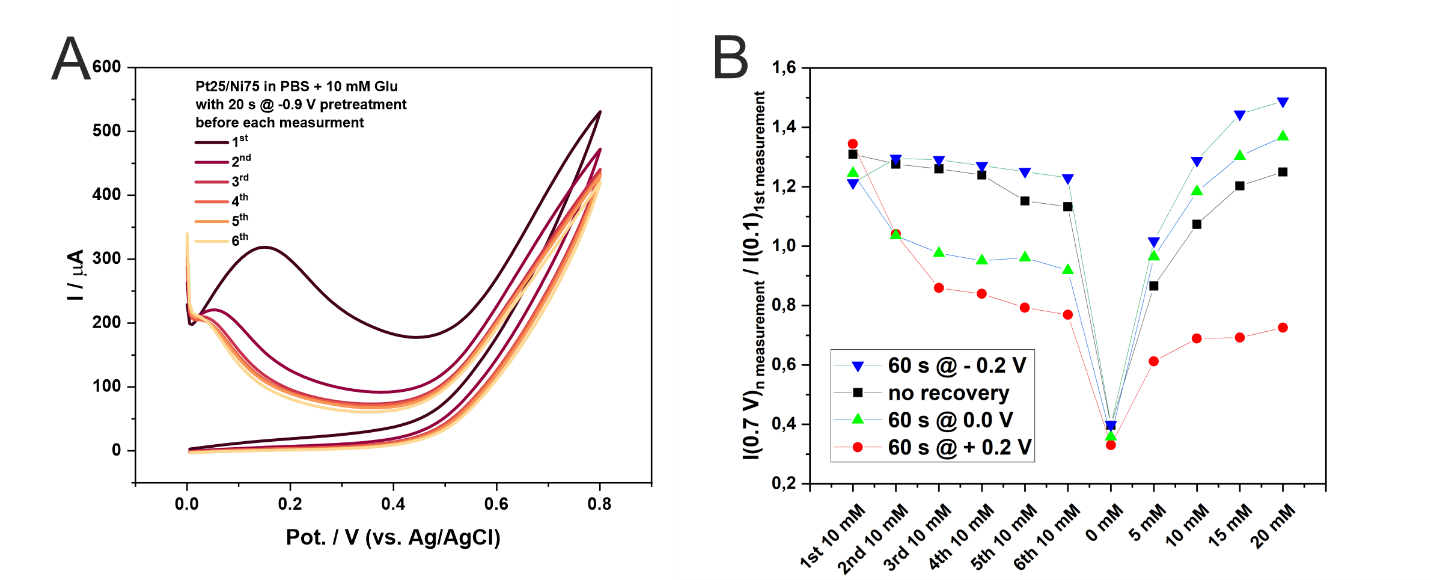
**

**Figure S13.** (A) Reusing an electrode for several consecutive measurements in PBS + 10 mM glucose solution. After each CV measurement, a fresh drop of solution was placed. (B) Impact of an electrode cleaning. The procedure was: pretreatment (-0.9 V for 20 s) - measurement (CV) - recovery treatment (-0.2/0.0/+0.2 V for 60 s) – place fresh drop on electrode. After 6 consecutive measurements in 10 mM glucose solution, a 0 mM, 5 mM, 10 mM, 15 mM, and 20 mM glucose in PBS was measured. For a better comparison between the electrodes, the ratio I(0.7 V)/I(0.1 V of first measurement) was used.

**7. Measurement in undiluted and diluted human serum**

**
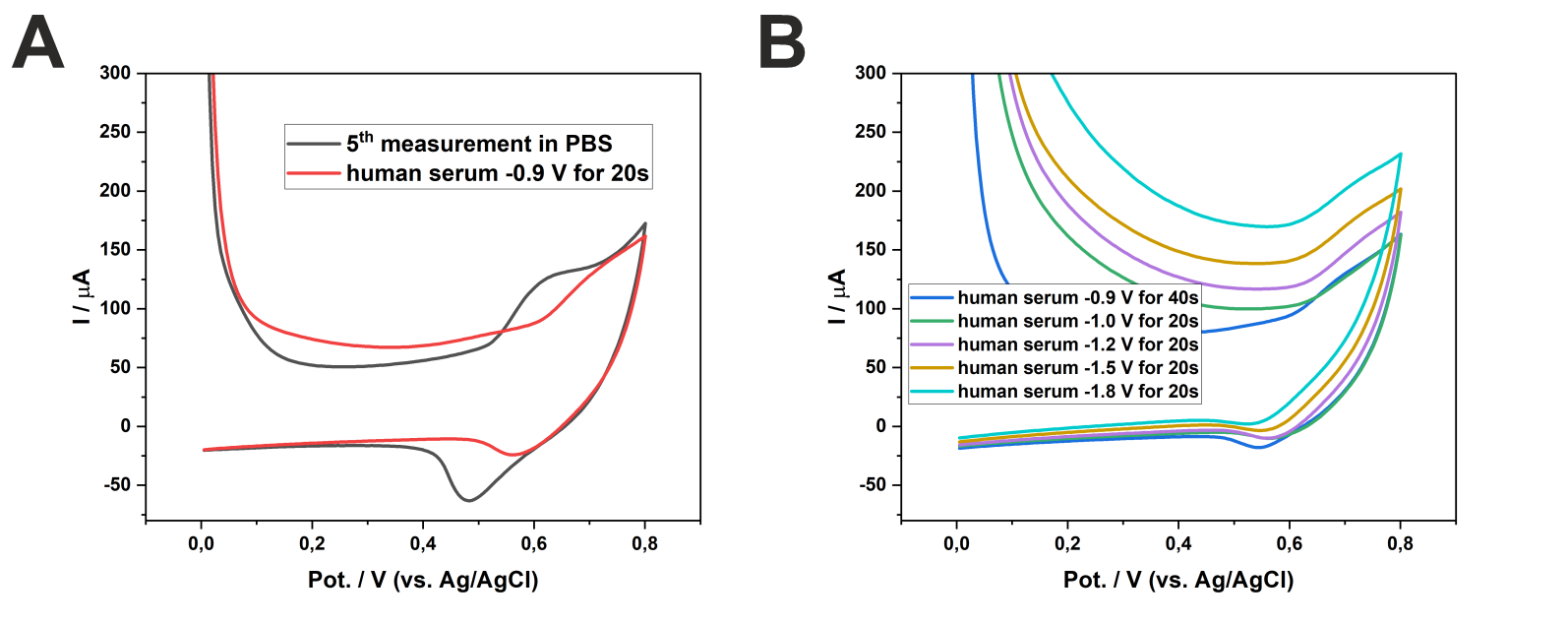
**

**Figure S14.** (A) The stabilized, 5^th^ measurement in PBS and a consecutive measurement in undiluted human serum. (B) CVs with various pretreatments in undiluted human serum.

**
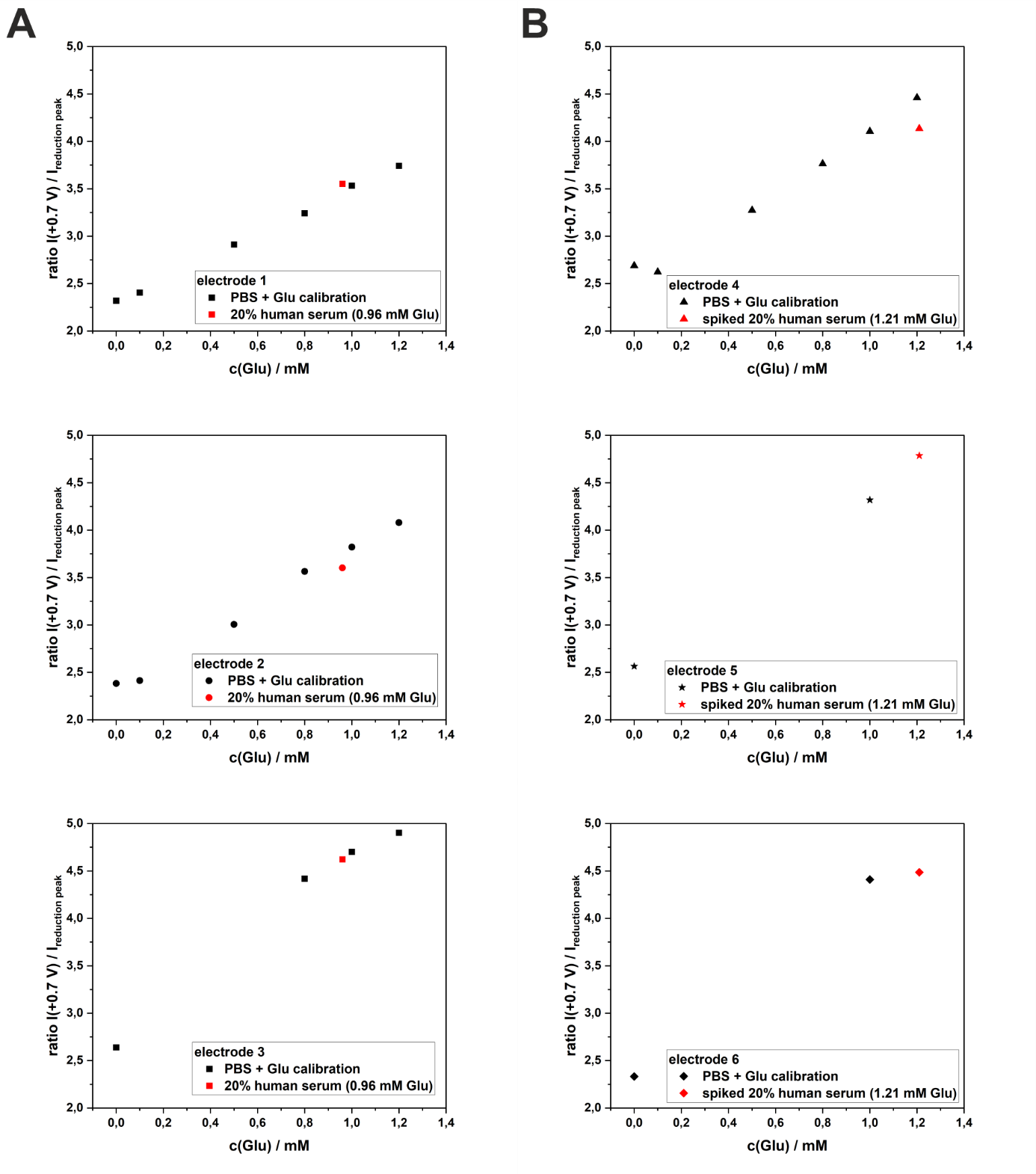
**

**Figure S15**. Calibration and measurements of (A) 20 % human serum (0.96 mM glucose according to Roche AccuChec) and (B) 20% human serum spiked with 0.25 mM glucose (in total 1.21 mM glucose. Due to good linearity of electrodes 1, 2 and 4, calibration measurements were reduced for electrodes 3, 5 and 6.

**Table S1** Comparison of non-enzymatic glucose sensors at physiological pH.

| **Electrode** | **Electrode fabrication technique**  **(*complexity*)** | **Electrochemical measurement** | **Detection limit (**μM) | **Linear range (**μM) | **Reference** |
| --- | --- | --- | --- | --- | --- |
| Carbon fiber microelectrode modified with Au and Ni nanoparticles | Electrochemical deposition of Au and Ni  (*high*) | *- in situ* generation of OH^-^ at −1.5 V, 20s  - amperometric detection at 0.5 V (stagnant) | 11.3 | 50 - 700 | [17] |
| Au rod modified Nafion | Commercial electrode  (*low*) | *- in situ* generation of OH^-^ at −2.0 V, 20s  - amperometric detection at 0.2 V (stagnant) | 15 | 30 -1100 | [18] |
| Dendritic gold nanostructures (AuNs) resembling feather branches electrodeposited onto a laser-scribed 3D graphene electrode (LSGE) | Electrodeposition of Au Nanostructures on LSGE  (*moderate*) | - amperometric detection at 0.2 V (stirred) | 210 | 500 - 20000 | [19] |
| Pd nanoparticle-encapsulated Co-based zeolitic imidazolate framework (Pd@ZIF-67) | Synthesis of Pd@ZIF-67, Screen printing  (*high*) | *- in situ* generation of OH^-^ at −2.0 V, 20s  - amperometric detection at 0.6 V (stagnant) | 2.0 | 10 – 1000 | [20] |
| Porous platinum black deposited on stainless-steel micro-needle electrode | Electrodeposition of porous platinum black  (*moderate*) | -amperometric detection at 0.12 V (stirred) | 268 | 1000 – 30000 | [21] |
| Au nanoflower coated carbon cloth (Au NFs@CC) | Adsorption of gold-seeds on carbon cloth, growth of Au NFs  (*moderate*) | - amperometric detection at 0.35 V (stirred) | 5.18 | 8 – 4000 | [22] |
| Au microspheres electrodeposited carbon cloth | Electrodeposition of gold on carbon cloth  (l*ow*) | - amperometric detection at 0.3 V (stirred) | 0.78 | 1 – 114  114 – 2164 | [23] |
| Au nanowrinkles @ reduced graphene oxide (rGO)/polyurethane composite fiber | Wet spinning, Cr-deposition by e-beam and Au layer deposition by thermal evaporation  (high) | - amperometric detection at 0.2 V (stirred) | 0.5 | 3 – 1710 | [24] |
| Pt/Ni LCNF | Electrospinning, laser scribing  (*low*) | *- in situ* generation of OH^-^ at −0.9 V, 20s  - cyclovoltammetric detection (stagnant) | 300 | 100 – 4000 | This work |

References

[1] D.A. Shirley, High-Resolution X-Ray Photoemission Spectrum of the Valence Bands of Gold, Phys. Rev. B 5 (1972) 4709–4714.

[2] J.H. Scofield, Hartree-Slater subshell photoionization cross-sections at 1254 and 1487 eV, Journal of Electron Spectroscopy and Related Phenomena 8 (1976) 129–137.

[3] F.T. Johra, J.-W. Lee, W.-G. Jung, Facile and safe graphene preparation on solution based platform, Journal of Industrial and Engineering Chemistry 20 (2014) 2883–2887.

[4] G. Wei, J. Yu, M. Gu, T.B. Tang, Dielectric relaxation and hopping conduction in reduced graphite oxide, Journal of Applied Physics 119 (2016).

[5] X. Chen, X. Wang, de Fang, A review on C1s XPS-spectra for some kinds of carbon materials, Fullerenes, Nanotubes and Carbon Nanostructures 28 (2020) 1048–1058.

[6] S. Rani, C. Byron, A.V. Teplyakov, Formation of silica-supported platinum nanoparticles as a function of preparation conditions and boron impregnation, The Journal of chemical physics 152 (2020) 134701.

[7] H.-W. Chang, Y.-C. Tsai, C.-W. Cheng, C.-Y. Lin, P.-H. Wu, Preparation of platinum/carbon nanotube in aqueous solution by femtosecond laser for non-enzymatic glucose determination, Sensors and Actuators B: Chemical 183 (2013) 34–39.

[8] W. Liu, Q. Chen, Y. Huang, D. Wang, L. Li, Z. Liu, In situ laser synthesis of Pt nanoparticles embedded in graphene films for wearable strain sensors with ultra-high sensitivity and stability, Carbon 190 (2022) 245–254.

[9] A. Scroccarello, R. Álvarez-Diduk, F. Della Pelle, C. de Carvalho Castro E Silva, A. Idili, C. Parolo, D. Compagnone, A. Merkoçi, One-Step Laser Nanostructuration of Reduced Graphene Oxide Films Embedding Metal Nanoparticles for Sensing Applications, ACS sensors 8 (2023) 598–609.

[10] R. Jiménez-Pérez, J. Agrisuelas, A. Gomis-Berenguer, M.T. Baeza-Romero, E. Valero, One-pot electrodeposition of multilayered 3D PtNi/polymer nanocomposite. H2O2 determination in aerosol phase, Electrochimica Acta 461 (2023) 142683.

[11] Z. Xi, K. Wei, Q. Wang, M.J. Kim, S. Sun, V. Fung, X. Xia, Nickel-Platinum Nanoparticles as Peroxidase Mimics with a Record High Catalytic Efficiency, Journal of the American Chemical Society 143 (2021) 2660–2664.

[12] H. Guan, Y. Zhao, J. Zhang, Y. Liu, S. Yuan, B. Zhang, Uniformly dispersed PtNi alloy nanoparticles in porous N-doped carbon nanofibers with high selectivity and stability for hydrogen peroxide detection, Sensors and Actuators B: Chemical 261 (2018) 354–363.

[13] P. Liu, Y. Zhang, L. Ye, M. Huang, T. Zeng, J. Yang, F. Tian, Z. Wu, X. Zhang, C. Hu, N. Yang, Laser-induced graphene decorated with Ni Pt alloy nanoparticles for non-enzymatic electrochemical quantification of glucose, Diamond and Related Materials 146 (2024) 111205.

[14] T. Hu, P. Li, W. Zhang, Y. Ye, J. Liu, Y. Cai, G. Zhang, K. Dai, C. Liang, Laser irradiation induced platinum-based bimetallic alloy nanoparticles in liquids for electrocatalytic hydrogen production, Journal of Alloys and Compounds 934 (2023) 167914.

[15] R. Siburian, H. Sihotang, S. Lumban Raja, M. Supeno, C. Simanjuntak, New Route to Synthesize of Graphene Nano Sheets, Orient. J. Chem 34 (2018) 182–187.

[16] G. Yasin, M. Arif, M. Shakeel, Y. Dun, Y. Zuo, W.Q. Khan, Y. Tang, A. Khan, M. Nadeem, Exploring the Nickel–Graphene Nanocomposite Coatings for Superior Corrosion Resistance: Manipulating the Effect of Deposition Current Density on its Morphology, Mechanical Properties, and Erosion‐Corrosion Performance, Adv Eng Mater 20 (2018).

[17] M. Abbasnia Tehrani, S.H. Ahmadi, S. Alimohammadi, P. Sasanpour, N. Batvani, S.H. Kazemi, M.A. Kiani, Continuous glucose monitoring using wearable non-enzymatic sensors in a physiological environment, Biosensors and Bioelectronics: X 18 (2024) 100482.

[18] X. Zhu, Y. Ju, J. Chen, D. Liu, H. Liu, Nonenzymatic Wearable Sensor for Electrochemical Analysis of Perspiration Glucose, ACS sensors 3 (2018) 1135–1141.

[19] A. Berni, A. Amine, J.J. García-Guzmán, L. Cubillana-Aguilera, J.M. Palacios-Santander, Feather-like Gold Nanostructures Anchored onto 3D Mesoporous Laser-Scribed Graphene: A Highly Sensitive Platform for Enzymeless Glucose Electrochemical Detection in Neutral Media, Biosensors 13 (2023).

[20] X. Zhu, S. Yuan, Y. Ju, J. Yang, C. Zhao, H. Liu, Water Splitting-Assisted Electrocatalytic Oxidation of Glucose with a Metal-Organic Framework for Wearable Nonenzymatic Perspiration Sensing, Analytical chemistry 91 (2019) 10764–10771.

[21] Y.-J. Kim, S.R. Chinnadayyala, H.T.N. Le, S. Cho, Sensitive Electrochemical Non-Enzymatic Detection of Glucose Based on Wireless Data Transmission, Sensors (Basel, Switzerland) 22 (2022).

[22] Z. Zhao, T. Wang, K. Li, D. Long, J. Zhao, F. Zhu, W. Gong, A flexible nonenzymatic sweat glucose sensor based on Au nanoflowers coated carbon cloth, Sensors and Actuators B: Chemical 388 (2023) 133798.

[23] Q. Peng, Y. Zhang, S. Yang, T. Yuwen, Y. Liu, J. Fan, G. Zang, Glucose determination behaviour of gold microspheres-electrodeposited carbon cloth flexible electrodes in neutral media, Analytica chimica acta 1159 (2021) 338442.

[24] P.T. Toi, T.Q. Trung, T.M.L. Dang, C.W. Bae, N.-E. Lee, Highly Electrocatalytic, Durable, and Stretchable Nanohybrid Fiber for On-Body Sweat Glucose Detection, ACS applied materials & interfaces 11 (2019) 10707–10717.
